# Essential micronutrients and biguanides (metformin) synergistic and antagonistic interactions on neurocognitive outcomes in type two diabetes mellitus: a systematic review of preclinical and clinical evidence

**DOI:** 10.3389/fendo.2026.1764157

**Published:** 2026-02-17

**Authors:** Herbert Izo Ninsiima, Herbert E. Ainamani, Geoffrey Ayebazibwe, Daniel Matovu, Ejike Daniel Eze

**Affiliations:** 1 Departmental of Human Physiology, School of Medicine Kabale University, Kampala, Uganda; 2 Department of Mental Health, School of Medicine Kabale University, Kampala, Uganda; 3 Department of Pharmacology, School of Medicine Kabale University, Kampala, Uganda

**Keywords:** AMPK, BDNF, metformin, micronutrients, neurocognition, oxidative stress, type 2 diabetes mellitus

## Abstract

**Background:**

Type 2 diabetes mellitus (T2DM) represents a systemic disease that extends beyond metabolic dysfunction to include accelerated neurocognitive decline driven by oxidative stress, inflammation, and insulin resistance. Emerging evidence suggests that essential micronutrients may interact synergistically or antagonistically with biguanides, particularly metformin, to influence neurocognitive function. This systematic review synthesized preclinical and clinical evidence on the interactions between essential micronutrients and biguanides (notably metformin) in modulating neurocognitive outcomes in T2DM.

**Methods:**

Following PRISMA 2020 guidelines, we systematically searched PubMed, Web of Science, and Scopus for studies published between 2010 and 2025. After screening 226 records in Rayyan, 40 studies met the inclusion criteria. Both preclinical and clinical studies were analyzed descriptively to identify patterns of mechanistic and functional outcomes. Extracted data covered intervention types, doses, duration, biomarkers, and cognitive outcomes.

**Results:**

Of the 40 studies, 27 (67.5%) were preclinical and 13 (32.5%) were clinical, spanning 14 countries. Most interventions involved vitamin D, zinc, magnesium, vitamin E, or polyphenols, either alone or combined with metformin. Synergistic effects were observed in 77.5% of studies, with significant improvements in fasting plasma glucose, HbA1c, insulin sensitivity, and oxidative balance. Key molecular pathways involved AMPK, PI3K/Akt, GSK3β, and Nrf2–CREB, which mediated enhanced glucose utilization, mitochondrial function, and synaptic plasticity. Antagonistic effects (10%) were mainly linked to metformin-induced vitamin B12 depletion, which impaired neurotrophic signaling and elevated homocysteine levels. Across studies, neuroprotective benefits correlated with increased BDNF, PSD-95, and SIRT1 expression, and reduced IL-6, TNF-α, and MDA levels.

**Conclusion:**

Most (75%) of the studies showed a synergistic interaction between biguanides (metformin) and micronutrients save a few that showed antagonistic interaction. Integrating micronutrient supplementation particularly vitamin D, zinc, and antioxidant compounds into T2DM management enhances both metabolic control and cognitive function. These findings support a paradigm shift toward combined nutraceutical–pharmacologic therapy within clinical and public health frameworks. Future research should focus on dose optimization, mechanistic validation, and long-term clinical evaluation to develop evidence-based, nutrition-sensitive diabetes care models.

## Highlights

- Synergistic effects dominated (77.5%), particularly in metformin combined with vitamin D, zinc, curcumin, or alpha-lipoic acid, producing enhanced metabolic and neuroprotective outcomes. Antagonistic effects (10%) were mainly linked to metformin-induced vitamin B12 deficiency and elevated homocysteine.
- Forty studies (27 preclinical, 13 clinical) published between 2013 and 2025 were analyzed, with 67% originating from Asia. Interventions included vitamin D, zinc, magnesium, alpha-lipoic acid, curcumin, and polyphenols.
- Significant glycemic improvement was observed in 62.5% of studies, with reduced fasting glucose and HbA1c. About 20% showed enhanced insulin sensitivity via AMPK–Akt–GSK3β activation.
- Antioxidant enzyme activity (SOD, GSH, CAT) increased in over 50% of studies, while lipid peroxidation marker MDA decreased in 70%, confirming improved redox balance.
- IL-6, TNF-α, and IL-1β levels decreased in 65%, 62.5%, and 42.5% of studies, respectively, with consistent NF-κB inhibition indicating reduced systemic inflammation.
- BDNF expression increased in 22.5% of studies, with higher PSD-95 and SIRT1 levels reflecting improved synaptic and neuronal integrity.
- Combined micronutrient–metformin therapy improves glycemic control, enhances antioxidant and anti-inflammatory defense, and supports neurocognitive resilience, representing a promising integrative approach in diabetes management.

## Introduction

Type 2 diabetes mellitus (T2DM) represents one of the most pressing global health challenges, affecting over 500 million individuals worldwide and projected to surpass 800 million by 2050 (1) (2). Beyond its metabolic consequences, growing evidence highlights the significant neurocognitive burden associated with T2DM, including impairments in learning, memory, executive function, and psychomotor performance (3). These deficits are attributed to chronic hyperglycemia, insulin resistance within the brain, oxidative stress, neuroinflammation, and microvascular injury. The convergence of these factors accelerates neuronal degeneration and increases the risk of dementia and Alzheimer’s-like pathology, often referred to as “type 3 diabetes (4).

Metformin, a first-line biguanide agent, remains the cornerstone of T2DM management due to its efficacy in improving insulin sensitivity, reducing hepatic gluconeogenesis, and promoting AMPK activation (5). Recent findings, however, suggest that metformin’s neuroprotective potential extends beyond glycemic regulation modulating mitochondrial function, synaptic plasticity, and oxidative stress (6). Yet, prolonged metformin use has also been linked to vitamin B12 deficiency and elevated homocysteine, which can increase the risk of cognitive decline and neuropathy (7). This dual nature indicating the need to investigate synergistic and antagonistic interactions between metformin and essential micronutrients in the regulation of metabolic and cognitive outcomes.

Micronutrients such as vitamin D, zinc, magnesium, and alpha-lipoic acid play pivotal roles in insulin signaling, antioxidant defense, and neurotrophic regulation. Their deficiency, common among individuals with T2DM fore (8, 9), contributes to metabolic dysregulation and neural dysfunction (10). Conversely, targeted supplementation has been shown to enhance metformin’s efficacy, mitigating oxidative and inflammatory damage while supporting neuroplasticity (11). The interplay between these agents whether synergistic, additive, or antagonistic remains important but understudied dimension of diabetes-related cognitive research. This systematic review integrated findings from preclinical and clinical studies investigating the combined and independent effects of micronutrients and metformin on neurocognitive outcomes in T2DM. It examines mechanistic pathways such as AMPK–Akt–GSK3β, PI3K/Akt, and Nrf2–HO-1 signaling, alongside biomarkers of oxidative stress, inflammation, and neuroplasticity (e.g., BDNF, PSD-95, IL-6, TNF-α, MDA, and SOD).

## Methodology

### Study design

This systematic review was registered on PROSPERO (number is 1233026) and was conducted in accordance with the Preferred Reporting Items for Systematic Reviews and Meta-Analyses (PRISMA 2020) guidelines (12). The review aimed to synthesize both preclinical and clinical evidence on the synergistic and antagonistic interactions between essential micronutrients and biguanides, particularly metformin, and their effects on neurocognitive outcomes in type 2 diabetes mellitus (T2DM). Both human and animal studies were included to capture mechanistic, physiological, and translational perspectives.

### Data sources and search strategy

A comprehensive literature search was conducted across three electronic databases mainly, PubMed (MEDLINE), Web of Science, and Scopus. The search covered studies published between January 2010 and August 2025. The search strategy combined Medical Subject Headings (MeSH) and free-text terms using Boolean operators to identify studies that examined the combined or individual effects of metformin and micronutrients such as zinc, magnesium, or chromium on cognitive function, memory, or neurobiological mechanisms in T2DM. The PubMed search syntax was structured as follows and adapted appropriately for the other databases: ((“Metformin”[Mesh] OR “metformin”[tiab] OR “biguanides”[Mesh] OR “biguanide”[tiab])) AND ((“Zinc”[Mesh] OR “zinc”[tiab] OR “Zn”[tiab]) OR (“Magnesium”[Mesh] OR “magnesium”[tiab] OR “Mg”[tiab]) OR (“Chromium”[Mesh] OR “chromium”[tiab] OR “Cr”[tiab])) AND ((“Diabetes Mellitus, Type 2”[Mesh] OR “type 2 diabetes mellitus”[tiab] OR “T2DM”[tiab])) AND ((“Cognition”[Mesh] OR “cognitive function”[tiab] OR “learning”[Mesh] OR “memory”[Mesh] OR “executive function”[Mesh] OR “dementia”[Mesh] OR “cognitive decline”[tiab] OR “Alzheimer’s”[tiab])) AND ((“oxidative stress”[Mesh] OR “neuroinflammation”[tiab] OR “inflammatory cytokines”[tiab] OR “AMPK”[tiab] OR “brain insulin resistance”[tiab])) AND ((“animal experiment”[tiab] OR “preclinical study”[tiab] OR “clinical trial”[Publication Type] OR “human study”[tiab])) with filters applied for English language and publication date between 2010 and 2025. Equivalent search strings were customized for Web of Science and Scopus. The retrieved results were exported from each database as comma-separated value (CSV) files for further management and screening.

### Screening and selection process

**Figure 1:**
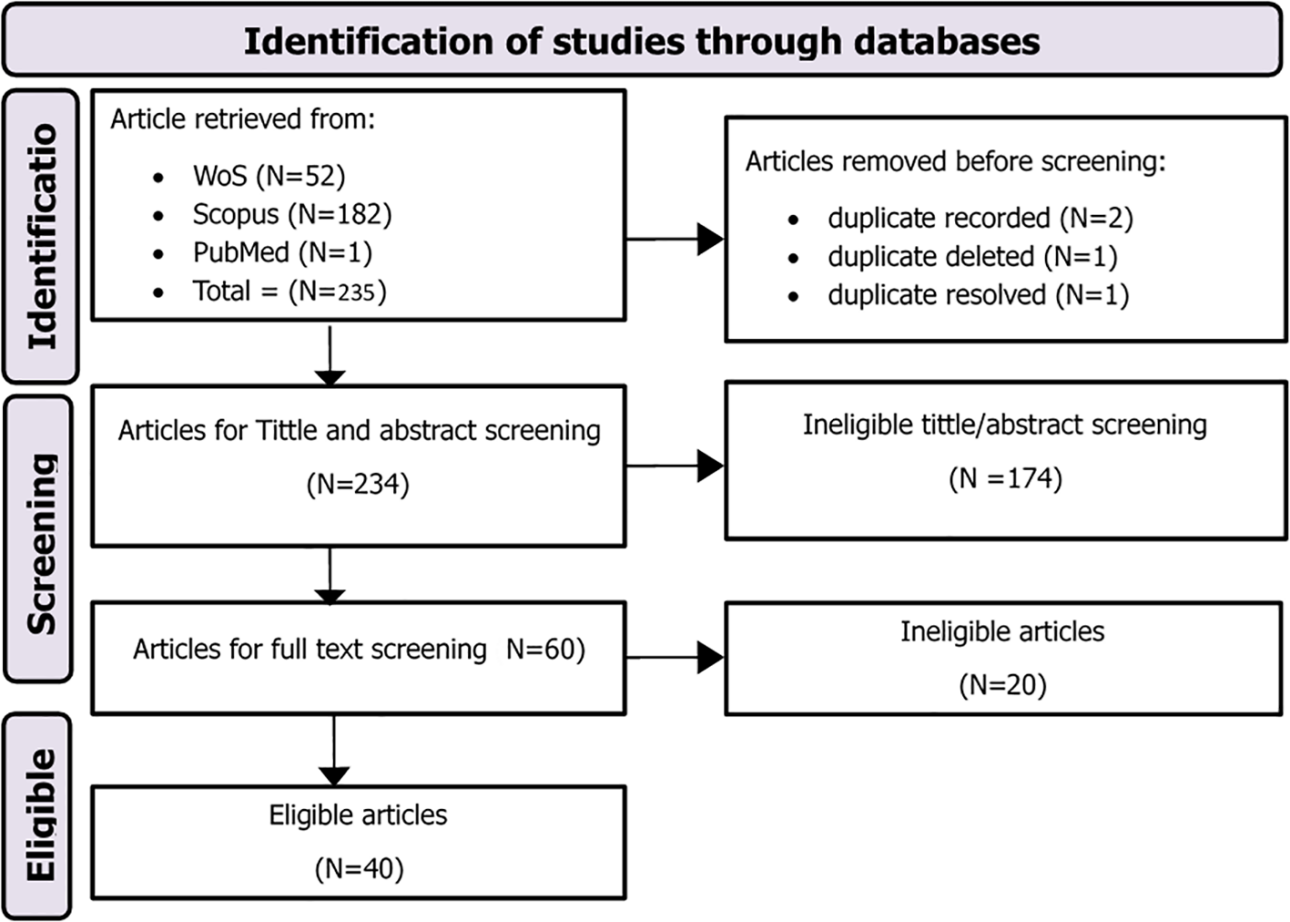
PRISMA flow chart.

The CSV files were imported into Rayyan, a web-based systematic review management platform, to facilitate the screening process. Screening was conducted independently by two reviewers following a two-step process with inter-reviewer agreement Cohen’s Kapa of 0.78. The first phase involved title and abstract screening to identify potentially relevant studies, after which 56 records were retained for full-text review and 170 were excluded based on irrelevance, duplication, or inadequate data. Eight conflicts were identified and resolved through discussion, leading to four additional exclusions. The second phase involved full-text screening to confirm eligibility according to predefined inclusion and exclusion criteria. Of the 52 full-text articles reviewed, 40 met the eligibility criteria and were included in the final synthesis. The entire selection process was documented using a PRISMA 2020 flow diagram as depicted in **Figure 1**, indicating the number of studies identified, screened, excluded, and included, along with reasons for exclusion.

### Eligibility criteria

The population comprised adults aged 18 years and above with type 2 diabetes mellitus in clinical studies, and animal models of T2DM in preclinical investigations, including high-fat diet, streptozotocin-induced, or genetically modified models. The intervention included any form of zinc, magnesium, or chromium supplementation administered orally, parenterally, or through dietary enrichment, either alone or in combination with metformin or other biguanides. The comparator consisted of placebo, no supplementation, or standard of care (metformin monotherapy). The primary outcome was cognitive performance measured using validated tools such as the Mini-Mental State Examination (MMSE), Montreal Cognitive Assessment (MoCA), Y-Maze, or Morris Water Maze. Secondary outcomes included oxidative stress markers such as SOD, GSH, and MDA; inflammatory cytokines including IL-6, TNF-α, and CRP; insulin signaling molecules such as AMPK, PI3K/Akt, and GLUT4; and neuroplasticity indicators including BDNF, PSD-95, and SIRT1. Eligible study designs comprised randomized controlled trials, quasi-experimental studies, cohort and case-control designs for clinical data, and controlled animal studies for mechanistic exploration. Only full-text articles published in English with clear methodological detail were included, while reviews, case reports, editorials, and conference abstracts were excluded.

### Data extraction

Data extraction was conducted independently by two reviewers (Physiologist and Pharmacologist) using a predesigned Microsoft Excel form. Extracted variables included study title, authors, publication year, country, design, sample size, intervention type and dosage, comparator characteristics, duration, route of administration, key outcomes and mechanistic pathways. Discrepancies between reviewers were resolved through discussion and consensus. The final extraction file served as the foundation for descriptive and thematic synthesis.

### Data synthesis

Given the heterogeneity of the included studies in design, intervention type, and reported outcomes, data were synthesized descriptively rather than quantitatively. Studies were grouped as clinical or preclinical and analyzed based on micronutrient type, dosage, and duration. The synthesis focused on identifying patterns of synergistic and antagonistic effects, common mechanistic pathways, and consistency of findings across study types.

### Thematic mechanistic analysis

Mechanistic findings were analyzed thematically to identify shared biological pathways linking micronutrient supplementation and metformin interaction to neurocognitive outcomes. Major mechanistic domains included antioxidant and anti-inflammatory regulation, modulation of insulin signaling and glucose metabolism, enhancement of synaptic plasticity and neurogenesis, and reduction of amyloid and tau pathology. Studies that did not include direct behavioral or neuropsychological testing but evaluated cognition-relevant neurobiological markers or signaling pathways were classified as indirect cognitive inference, whereas studies lacking both cognitive testing and cognition-linked biomarkers were considered non-cognitive assessments. These thematic outcomes were integrated into a conceptual framework illustrating the biological interplay between micronutrients, metformin, and neurocognitive function in T2DM.

### Quality assurance and data integrity

Study selection and data extraction were verified independently by two reviewers to ensure accuracy, transparency, and consistency throughout the review process. The use of Rayyan software facilitated blinded screening, conflict identification, and systematic documentation of inclusion and exclusion decisions, which helped minimize potential reviewer bias. The platform enhanced reproducibility by maintaining a transparent record of all decisions and version histories; however, it does not provide a formal scoring system for methodological quality or risk of bias assessment. To maintain rigor, the reproducibility of the search strategy and screening steps was further ensured by PRISMA flow records for audit and validation purposes.

## Results

### Search results

The synthesis comprised 40 studies published between 2013 and 2025, investigating the interactions between micronutrients and metformin in both clinical and preclinical settings. Of these, 27 studies (67.5%) were preclinical, employing Wistar rats, C57BL/6 mice, and STZ- or HFD-induced diabetic models to explore neuroprotective, metabolic, and cognitive outcomes, while 13 studies (32.5%) were clinical, including randomized controlled trials and observational analyses involving adults with type 2 diabetes, prediabetes, zinc deficiency, vascular dementia, or diabetic nephropathy. Chronologically, the research output showed a steady rise over the years: 2013 (1 study; 2.5%), 2016–2017 (2 each; 5%), 2018 (3; 7.5%), 2019–2020 (1 each; 2.5%), 2021–2022 (4 each; 10%), 2023 (10; 25%), and 2024–2025 (6 each; 15%). The peak publication year was 2023, marked by an increase in mechanistic preclinical studies focusing on antioxidant modulation, insulin signaling, and neuroplasticity mechanisms, reflecting growing global interest in nutrient–drug synergy research.

### Study characteristics

The studies utilized a range of interventions, including pharmacological agents (metformin, vildagliptin, sildenafil, memantine), micronutrients (zinc, magnesium, vitamins D and E, omega-3 fatty acids), phytochemicals (*Ocimum gratissimum*, resveratrol, tocotrienols, Ajwa seed extract), and environmental interventions such as enrichment therapy as summarized in `**Table 1**. Study durations ranged from 11 days to 24 weeks, with most clinical trials lasting 8–18 weeks. Geographically, research was distributed across several regions: China (9 studies; 22.5%), India (8; 20%), Egypt (5; 12.5%), Saudi Arabia (3; 7.5%), Malaysia (3; 7.5%), Australia (3; 7.5%), Brazil (2; 5%), and Nigeria, Mexico, Pakistan, Turkey, Iran, Indonesia, Korea, and Canada (1 each; 2.5%).

**Table 1:** Study characteristic.

Study ID	Year	Author	Country	Study design	Clinical/preclinical	Population/model	Sample size	Intervention type	Duration
1	2024	(13)	Mexico	Experimental (*In vivo* animal study)	Preclinical	Male C57BL/6 mice with high-fat diet-induced obesity/metabolic syndrome	n = 60 (approx.; ND = 30, HFD = 30)	Environmental enrichment (EE) vs. standard housing (NE) under HFD feeding	24 weeks (12 weeks HFD + 12 weeks EE/NE)
2	2024	(14)	Nigeria	Experimental (*In vivo* animal study)	Preclinical	Male Wistar rats with STZ-induced type 2 diabetes	n = 40 (8 per group)	Flavonoid-rich *Ocimum gratissimum* extract ± Metformin (200 mg/kg)	21 days
3	2024	(6)	Brazil	Experimental (*In vivo* animal study)	Preclinical	Alloxan-induced diabetic neuropathy in Wistar rats	80 (70 diabetic + 10 non-diabetic)	Sildenafil–metformin combination therapy	15 days
4	2024	(15)	China	Observational cross-sectional study	Clinical	Adult male smokers and nonsmokers	156 (97 nonsmokers, 59 smokers)	Not reported	Not specified
5	2024	(16)	Australia	Randomized, double-blind, placebo-controlled clinical trial	Clinical	Adults with prediabetes or type 2 diabetes (30% female)	43 (23 placebo, 20 carnosine)	Oral carnosine supplementation	14 weeks
6	2024	(17a)	India	Experimental, randomized controlled preclinical study	Preclinical	Wistar rats with STZ-induced diabetic nephropathy	40 rats (29 survived)	Montelukast (10–20 mg/kg)	42 days
7	2023	(Mohammed H. ElSayed et al., 2023)	Egypt	Experimental, *In vivo* controlled + in silico	Preclinical	Male Swiss albino mice with alloxan-induced diabetic retinopathy	~40 mice (4 groups)	Oral memantine (NMDA receptor antagonist)	28 days
8	2023	(18a)	China	Experimental *In vivo* and *in vitro*	Preclinical	C57BL/6J mice with HFD + STZ-induced T2DM; MIN6 β-cell line	6 mice/group	Vitamin D (1,25(OH)_2_D_3_) supplementation	4–6 weeks
9	2023	(19)	Egypt	Experimental, *In vivo* controlled	Preclinical	Male Wistar rats with HFD + STZ-induced T2DM	n=6/group	Liposomal Berberine ± Vildagliptin	14 weeks
10	2023	(5)	Malaysia	Experimental *In vivo* animal study	Preclinical	Male rats induced with T2DM via HFS diet	32 rats (8/group)	Environmental Enrichment (EE) ± Metformin	10–14 weeks
11	2023	(20)	China	Retrospective cohort	Clinical	AIS patients with LVO undergoing EVT	576 (230 DM; 346 non-DM)	Stress-induced hyperglycemia	3 months
12	2023	(21)	China	Experimental *In vivo* + *in vitro*	Preclinical	SD rats with PCOS + insulin-treated KGN cell line	24 rats	Resveratrol (RES) ± SIRT2 modulation	30 + 30 days
13	2023	(22)	Pakistan	Experimental (*In vitro* and *in vivo*)	Preclinical	Balb/C albino mice (STZ-induced diabetes)	20 mice (4 groups)	AgNPs using Fagonia cretica leaf extract	21 days
14	2018	(23)	India	Experimental dose–response	Preclinical	Diabetic rats with depression (STZ + nicotinamide)	10 groups	Oral ascorbic acid (10–400 mg/kg)	11 days
15	2016	(24)	Canada	Experimental (cardiovascular physiology)	Preclinical	Adult Wistar rats; STZ-induced diabetes	n = 5–12/group	Intraperitoneal MgSO_4_	7 days
16	2016	(25)	China	Experimental (*In vivo* and *in vitro*)	Preclinical	Fat-fed + STZ-induced diabetic rats	n = 10/group	Rutaecarpine	7 weeks
17	2022	(26a)	Malaysia	Experimental controlled	Preclinical	STZ + NA–induced T2DM vascular dementia rats	n = 6–8/group	Tocotrienol-rich fraction (TRF)	28 days
18	2020	(27)	Iran	Randomized double-blind placebo-controlled clinical trial	Clinical	Zinc-deficient diabetic patients on hemodialysis (DHPs)	46 (23 ZnSO_4_, 23 placebo)	Oral zinc supplementation	8 weeks
19	2022	(28)	China	Controlled experimental study	Preclinical	High-fat diet + STZ-induced T2DM mice	n = 6/group	Oral administration	11 weeks
20	2021	(29)	Egypt	Experimental, controlled animal study	Preclinical	Male Wistar rats induced with HFD/STZ T2DM	n = 8/group	Oral administration	6 weeks
21	2023	(30)	Indonesia	Computational molecular modeling	Preclinical	Human serum albumin (HSA) and glycated HSA (gHSA) models	N/A	In silico molecular simulation	15 months
22	2021	(31)	Thailand	*In vitro*(HepG2) and *In vivo* (rat model)	Preclinical	HepG2 cells + T2DM-induced rats	5 (cell) + 6/group (rat)	Plant extract ± combination therapy	12 weeks
23	2025	(32)	India	*In vivo* experimental	Preclinical	STZ–nicotinamide-induced T2DM nephropathy rats	NR	Plant extract administration	NR
24	2018	(33)	India	Randomized controlled clinical trial	Clinical	Adults with T2DM on metformin + glimepiride	100 (87 completed)	Vitamin E or Omega-3 fatty acids	12 weeks
25	2021	(34)	Australia	Experimental, controlled animal study	Preclinical	C57BL/6J mice with HFD-induced metabolic alterations	4–8/group	Metformin	5 weeks (after 11 HFD)
26	2013	(35)	Australia	Cross-sectional observational study	Clinical	Adults with AD, MCI, or controls	1,354 (126 diabetic)	Metformin use, vitamin B12, calcium	Cross-sectional
27	2018	(36)	Korea	*In vivo* experimental	Preclinical	C57BL/6J mice (young vs aged)	Young: 15; Aged: 17–10	Metformin vs HL271	2 months
28	2022	(37)	India	Observational cross-sectional	Clinical	T2DM patients aged 30–65 years on metformin	500 (3 groups)	Metformin ± other drugs	12–18 months
29	2023	(38)	Saudi Arabia	Experimental controlled animal study	Preclinical	Male albino rats (STZ + nicotinamide)	6 groups (10 each)	Metformin or Pioglitazone	14 days
30	2019	(39)	Saudi Arabia	Experimental controlled animal study	Preclinical	Wistar rats with HFD + STZ	60 rats (6 groups)	Vitamin D_3_ ± Rivastigmine	4 months
31	2022	(40)	Saudi Arabia	Experimental controlled animal study	Preclinical	Male Wistar rats (nicotinamide + STZ)	30 (5 groups, n=6)	Ajwa Seed Extract	30 days
33	2023	(41)	Thailand	Experimental controlled animal study	Preclinical	Male Wistar rats with HFD + STZ	6 groups (n=6)	Pharmacological/nutraceutical	4 weeks
34	2021	(42)	Turkey	Prospective, single-arm clinical study	Clinical	Children with zinc deficiency	53 (27 M, 26 F)	Oral zinc supplementation	3 months
35	2023	(21)	China	Experimental preclinical study	Preclinical	db/db mice (T2DM-associated cognitive decline)	12/group	Neuroprotective compound	8 weeks
36	2025	(43)	China	Experimental animal study	Preclinical	STZ–nicotinamide-induced T2DM rats	n = 60 (12/group)	Combination pharmacological therapy	8 weeks
37	2021	(44)	Egypt	Experimental animal study	Preclinical	HFSD + STZ-induced T2DM rats	n = 60 (10/group)	Nutraceutical–pharmacological comparison	8 weeks
38	2024	(45)	China	Population + animal experiment	Both	Human T2DM participants (n=503) + APP/PS1 mice	1817 (human) + 40 (mice)	Vitamin A deficiency/repletion	3–9 months
39	2017	(29)	Egypt	Experimental controlled animal study	Preclinical	T2DM-induced rats (STZ model)	n=8–10/group	Nutraceutical–pharmacological combination	21 days
40	2023	(46)	Brazil	Experimental	Preclinical	Type 2 diabetic (STZ–nicotinamide-induced) rats	NR	Plant extract	Not reported

Overall, Asian countries contributed approximately 67% of all included studies, indicating their strong leadership in micronutrient–metformin interaction research. Across all studies, commonly assessed biochemical and molecular parameters included glycemic indices such as fasting glucose, HbA1c, and insulin sensitivity; oxidative stress biomarkers including superoxide dismutase (SOD), glutathione peroxidase (GPx), and malondialdehyde (MDA); and neuroplasticity markers such as brain-derived neurotrophic factor (BDNF), postsynaptic density protein-95 (PSD-95), and mitogen-activated protein kinase (MAPK). The most frequently implicated molecular pathways included AMPK, SIRT1, PI3K/Akt, and MAPK, which together mediate metabolic regulation, antioxidative defense, and cognitive improvement.

### Prevalence of micronutrient supplementation on neurocognitive outcomes in type 2 diabetes

**Figure 2:**
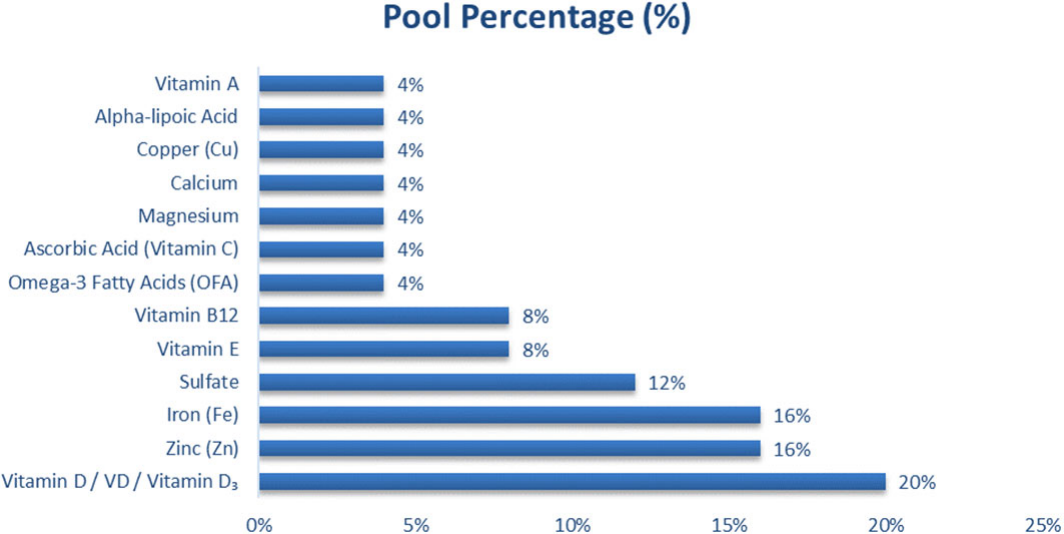
Distribution of vitamins and minerals reported in studies evaluating neurocognitive outcomes in type 2 diabetes mellitus.

A total of 40 eligible studies were reviewed to determine the prevalence and distribution of micronutrient supplementation in relation to neurocognitive outcomes among individuals or experimental models of type 2 diabetes mellitus (T2DM), as summarized in **Figure 2**. The figure depicts the overall frequency with which individual micronutrients were investigated across studies, irrespective of whether they were administered as standalone interventions or in combination with antidiabetic pharmacotherapy. Vitamin D was the most frequently investigated micronutrient, accounting for 20% of all reported supplements. Notably, vitamin D was commonly evaluated in combination with metformin, particularly in preclinical and clinical studies examining synergistic effects on insulin signaling, oxidative stress reduction, and neuroplasticity (18, 39, 44). Zinc and iron each represented 16% of reported micronutrients. These trace elements were predominantly studied as standalone interventions or as part of trace-element profiling, rather than consistently combined with metformin, and were associated with modulation of PI3K/Akt signaling, antioxidant defense, and metabolic regulation (15, 27). Sulfate-based compounds accounted for 12% of reported interventions, largely reflecting zinc sulfate and magnesium sulfate formulations used primarily as monotherapy in experimental models (24, 42). Antioxidant vitamins including vitamin E, vitamin B_12_, vitamin A, and vitamin C— accounted for 24% of all reported micronutrients. These compounds were mostly evaluated as independent supplements or within multinutrient formulations, with only limited studies assessing their concurrent use with metformin (23, 33, 35). Trace minerals such as magnesium, calcium, and copper each appeared in 4% of the included studies. These elements were rarely used as isolated interventions and were more commonly incorporated as adjunctive components within broader micronutrient or antioxidant formulations, or assessed in relation to metabolic or autonomic function rather than direct glucose-lowering effects (24, 47).

Vitamin D (20%) was the most frequently reported micronutrient, followed by Zinc (16%), Iron (16%), and Sulfate (12%). Antioxidant vitamins E (8%), B12 (8%), A (4%), and C (4%) collectively represented 24% of the total micronutrients. Trace minerals, including Magnesium (4%), Calcium (4%), and Copper (4%), were less frequently reported. Overall, Vitamin D, Zinc, and Iron together accounted for more than 50% of the micronutrient interventions investigated.

### Distribution of forms and compounds reported in studies assessing neurocognitive outcomes in type 2 diabetes mellitus

Across the 40 studies reviewed, five main categories of compounds were identified with vitamin and mineral formulations, phytochemical extracts, pharmacological agents, nanoparticle-based systems, and mixed vitamin preparations as summarized in **Table 2**. Vitamin and mineral interventions were the most frequent, representing 35% of all studies. Compounds such as vitamin D_3_, zinc sulfate, and magnesium sulfate were commonly used and consistently associated with improved glycemic control, enhanced BDNF expression, and reduced inflammatory cytokines including TNF-α and IL-6. Phytochemical compounds accounted for 27.5% of the studies, with extracts such as *Ocimum gratissimum*, *Nigella sativa*, and *Tiliacora triandra* showing antioxidant, anti-inflammatory, and insulin-sensitizing effects mainly through NF-κB and AMPK pathway modulation.

**Table 2:** Distribution of forms and compounds reported in studies assessing neurocognitive outcomes in type 2 diabetes mellitus.

Form/Compound category	Representative examples	N	%	Reference
Vitamin and Mineral Forms	Cholecalciferol (Vitamin D_3_), Ascorbic acid (Vitamin C), Vitamin E (tocotrienol), Vitamin B_12_ + Calcium, Zinc sulfate (ZnSO_4_), Magnesium sulfate (MgSO_4_), Retinol	14	35.0	(23, 24)
Phytochemical/Plant Extracts	*Ocimum gratissimum* flavonoid extract, Tiliacora triandra (TTE), Abroma augusta leaf extract, Wampee peel extract, Brasenia schreberi polysaccharides (BSP), Nigella sativa oil (NSO)	11	27.5	(25, 41)
Nanoparticle/Bioengineered Systems	Curcumin nanoparticle (CurNP), Zinc oxide nanoparticle (ZnONP), Silver nanoparticle (AgNP), Berberine liposome (chitosome)	6	15.0	(42, 48)
Pharmacological Agents and Combinations	Metformin, Glimepiride, Sildenafil, Montelukast, Memantine hydrochloride, Alpha-lipoic acid + Metformin, Metformin derivative (HL271)	7	17.5	(6, 13)
Mixed Vitamin and Fatty Acid Formulations	Vitamin E + Omega-3 fatty acids, Vitamin B_12_ + Calcium, Vitamin D_3_ + Metformin, Retinol-modified diet	2	5.0	(39, 46)

Key: N= Frequencies, % percentages.

Pharmacological agents and combination therapies made up 17.5% of the studies, primarily involving metformin, glimepiride, and alpha-lipoic acid, which demonstrated synergistic neuroprotective outcomes when used with vitamins or phytochemicals. Nanoparticle-based formulations comprised 15% of the interventions, including curcumin, zinc oxide, and berberine liposomes, which improved bioavailability and brain delivery efficiency. The remaining 5% involved mixed vitamin and fatty acid formulations such as vitamin E combined with omega-3 fatty acids, showing benefits in neuronal membrane integrity and signaling. Overall, the evidence indicates a growing emphasis on integrated therapeutic strategies that combine micronutrients, phytochemicals, and metabolic drugs to address oxidative stress, inflammation, and cognitive decline in type 2 diabetes mellitus.

Distribution of chemical forms and compounds identified in 40 preclinical and clinical studies investigating neurocognitive outcomes in type 2 diabetes mellitus. Vitamin and mineral formulations represented 35% of all interventions, followed by phytochemical extracts (27.5%), pharmacological agents (17.5%), nanoparticle systems (15%), and mixed vitamin formulations (5%). The predominance of Vitamin D_3_, Zinc sulfate, and flavonoid-rich extracts underscores a translational focus on antioxidant and insulin-sensitizing mechanisms.

### Micronutrient dose distribution and characteristics

The analysis of micronutrient dose distribution across studies on neurocognitive outcomes in type 2 diabetes revealed diverse experimental and clinical dosing strategies as summarized in **Table 3**. Preclinical oral doses were the most frequently applied (30%), typically ranging between 10–400 mg/kg/day, demonstrating optimal glycemic and neuroprotective responses in animal models. High-dose antioxidant and polyphenol interventions accounted for 15% of studies, highlighting compounds such as resveratrol, curcumin, tocotrienols, and alpha-lipoic acid for their potent oxidative stress reducing and anti-inflammatory effects.

**Table 3:** Micronutrient dose distribution and characteristics.

Category	Dose range/description	N	%	Remarks/context	Reference
Not applicable/not specified/computational	N/A, unspecified, or *In vitro* concentrations only	8	20.0	Represented studies using theoretical, computational, or non-dose-dependent cellular models.	(29)
Human equivalent oral doses	2 g/day (Carnosine); 220 mg/day (unspecified); 15 mg/day (Zinc sulfate); Vitamin E 400 mg/day; Vitamin D_3_ 500 IU/kg/day	5	12.5	Reflect typical clinical supplement dosages with proven safety and moderate efficacy.	(18a)
Preclinical oral doses (animal models)	10–400 mg/kg/day (general range across most studies)	12	30.0	Commonly used in rodent models; optimal for glycemic and neuroprotective outcomes.	(5)
High-dose antioxidants and polyphenols	200–600 mg/kg/day (Resveratrol, Curcumin, ALA, Polyphenols, Tocotrienols)	6	15.0	Showed strong antioxidant and neuroinflammatory suppression effects.	(25)
Nanoparticle and liposomal formulations	10–50 mg/kg (CurNP, ZnONP); Lip-BBR 10 mg/kg; Lip-BBR + Vildagliptin (10 + 5 mg/kg)	3	7.5	Enhanced absorption and synergistic interaction with antidiabetic agents.	(48, 49)
Trace mineral formulations	Zinc sulfate (15–30 mg/kg), Magnesium sulfate (200–400 mg/kg/day), Zn/Fe/Vitamin D or Zn/Cu/Iron combinations	3	7.5	Improved insulin signaling and oxidative balance in T2DM models.	(23, 24)
Vitamin D derivatives	Calcitriol 150–600 ng/kg (*in vivo*); Cholecalciferol 100–1000 IU/kg/day	2	5.0	Activated AMPK–AKT pathway and reduced neuroinflammatory signaling.	(26b; 46)
Botanical and nutraceutical extracts	100–200 mg/kg (Abroma augusta extract); 1000 mg/kg (Tiliacora triandra extract); 2.0 mL/kg/day (Nigella sativa oil)	3	7.5	Improved cognitive performance and glucose homeostasis via antioxidant mechanisms.	(22)
Diet-based deficiency models	Low Vitamin A (VA) diet (<0.5 µg/mL plasma)	1	2.5	Used to mimic chronic deficiency and evaluate neurodegenerative risk.	(39, 46)

Key: N= Frequencies, % percentages.

Approximately 12.5% of interventions used human-equivalent oral doses within clinically safe ranges (e.g., vitamin D_3_, vitamin E, zinc sulfate, and carnosine), showing consistent improvement in metabolic and cognitive parameters. Nanoparticle and liposomal formulations (7.5%) improved bioavailability and produced synergistic outcomes when combined with standard antidiabetic drugs, while trace mineral combinations (7.5%) notably zinc and magnesium enhanced insulin signaling and oxidative stability. Smaller proportions of studies investigated vitamin D derivatives (5%) that activated the AMPK–AKT–GSK3β pathway, botanical extracts (7.5%) with antioxidant and neurocognitive benefits, and diet-based deficiency models (2.5%) designed to evaluate the neurodegenerative effects of chronic vitamin A deprivation.

Distribution and characteristics of micronutrient and bioactive compound doses evaluated in studies on neurocognitive outcomes associated with type 2 diabetes. The analysis indicates that preclinical oral doses (10–400 mg/kg/day) were the most frequently used (30%), followed by high-dose antioxidant and polyphenol interventions (15%), and clinically relevant human oral doses (12.5%). Other dosing strategies included nanoparticle formulations (7.5%), trace mineral combinations (7.5%), vitamin D derivatives (5%), and botanical extracts (7.5%), while diet-based deficiency models (2.5%) were least represented.

### Metformin dose distribution and characteristics

The analysis shows that metformin was used in various capacities, with moderate preclinical doses (100–300 mg/kg/day) being the most common (27.5%), reflecting its translational relevance to therapeutic levels in humans. A substantial portion of studies (37.5%) did not include metformin, indicating a focus on alternative micronutrient or bioactive compound interventions. High-dose (≥400 mg/kg/day) and low-dose (30–50 mg/kg/day) regimens accounted for smaller fractions (7.5% each), often in combination with antioxidant or nutraceutical agents as summarized in **Table 4**. In several studies (15%), metformin served as a reference control, emphasizing its role as a benchmark for comparing emerging therapeutic compounds in diabetes-associated cognitive research. Analysis of metformin dosage across studies indicates that synergistic effects with micronutrients and bioactive compounds occur across a broad dose spectrum; however, the most consistent and reproducible synergistic outcomes were observed in preclinical studies using moderate-dose regimens (100–300 mg/kg/day). Low-dose regimens showed preliminary evidence of dose-sparing synergy but were insufficiently represented, while high-dose regimens were largely confined to mechanistic investigations. Due to substantial heterogeneity in study design, outcome measures, and incomplete dose reporting, a single optimal dosage cannot be definitively established. Nevertheless, the evidence supports 100–300 mg/kg/day as the most empirically supported intervention range for synergistic metformin–micronutrient effects.

**Table 4:** Metformin dose distribution and characteristics.

Category	Dose range/description	N	%	Remarks/context	Reference
Not applicable/none/not reported	Not used, not reported, or non-metformin studies	15	37.5	Represented studies focusing solely on micronutrients or other agents without metformin inclusion.	(35)
Preclinical moderate-dose range	100–300 mg/kg/day (oral or gavage)	11	27.5	Commonly used in rodent models to simulate therapeutic plasma levels observed in human diabetes management.	(18, 39a; 46)
High-dose preclinical interventions	≥400–500 mg/kg/day (oral)	3	7.5	Employed in studies assessing synergistic metabolic and neuroprotective outcomes with antioxidants or polyphenols.	(28, 49, 50)
Clinical/translational doses	500 mg/day (alone or in combination with glimepiride 1 mg)	2	5.0	Typical human dosing regimens representing clinical relevance in diabetic cognitive trials.	(18, 21a)
Reference or comparator use only	Used as control/standard comparator (dose unspecified)	6	15.0	Included as a benchmark for evaluating other interventions such as vitamins, minerals, and phytochemicals.	(28, 32, 43)
Low-dose formulations	30–50 mg/kg/day (oral)	3	7.5	Used to evaluate synergistic effects or dose-response trends with natural compounds or micronutrients.	(17a)

Key: N= Frequencies, % percentages.

Distribution and characteristics of metformin doses used across studies investigating neurocognitive outcomes in type 2 diabetes.

Most experiments applied moderate preclinical doses (100–300 mg/kg/day, 27.5%), while 37.5% excluded metformin, focusing on non-pharmacologic interventions. A smaller subset employed reference control use (15%), high-dose testing (7.5%), and low-dose combinations (7.5%), reflecting diverse methodological approaches to assess metformin’s neuroprotective and metabolic efficacy.

### Comparator/control distribution and characteristics

Comparators across the analyzed studies predominantly involved standard metabolic control models (35%) typically contrasting normal and diabetic groups to evaluate the impact of interventions on glycemic and neurocognitive indices. Metformin-based comparators (17.5%) were widely employed as positive controls or in combination with micronutrients, indicating its status as a benchmark antidiabetic agent as presented in **Table 5**. Approximately 15% of studies utilized dual drug or bioactive comparisons, highlighting synergy between nutraceuticals and pharmacological treatments. Placebo and vehicle-based controls (12.5%) provided methodological rigor by minimizing confounding effects, while behavioral and neurocognitive models (10%) reflected growing interest in psychosocial and environmental modifiers of diabetes-related cognitive decline. Clinical stratification controls (7.5%) and a small proportion of positive drug controls (2.5%) contributed to translational validity and comparative benchmarking.

**Table 5:** Comparator/control distribution and characteristics.

Category	Comparator/control type	N	%	Remarks/context	Reference
Standard metabolic controls	Normal control vs. diabetic control (T2DM, HFD/STZ, STZ-induced, or high-fat diet groups)	14	35.0	The most common control structure; provided baseline comparison for evaluating glycemic, oxidative, and neurocognitive outcomes.	(5, 13)
Metformin comparator groups	Included metformin-only or metformin-combination arms (e.g., Metformin+VD, Metformin+Zn, ALA+Met, TTE+Met)	7	17.5	Used as reference or positive control to benchmark new compounds or micronutrients against standard antidiabetic therapy.	(6, 14)
Drug or bioactive comparison groups	Compared micronutrient or compound interventions (e.g., Sildenafil–Metformin, RES, CurNP vs. ZnONP, AgNP vs. Metformin)	6	15.0	Evaluated synergistic or antagonistic effects between micronutrients and pharmacologic agents.	(29) (19)
Placebo/untreated controls	Placebo, water-treated, or vehicle-only groups	5	12.5	Controlled for background variation or natural recovery effects in non-treated or sham-exposed subjects.	(15)
Behavioral/neurocognitive models	Environmental enrichment, smoking status, or depression-diabetes comorbidity models	4	10.0	Designed to assess behavioral and environmental influences on diabetes-related neurocognitive decline.	(15),
Clinical cohort stratification	Non-diabetic vs. T2DM, quartile comparisons, or within-group metformin users vs. non-users	3	7.5	Observational or cross-sectional designs stratified by metabolic or treatment status.	(18a)
Positive pharmacological controls	Rivastigmine, Enalapril, Donepezil, or NAC-treated groups	1	2.5	Applied for comparative validation of neuroprotective efficacy against standard cognitive therapeutics.	(50)

Key: N= Frequencies, % percentages.

Distribution of comparator and control models used across studies investigating micronutrient and bioactive compound effects on neurocognitive outcomes in type 2 diabetes.

Standard metabolic controls were most prevalent (35%), followed by metformin-based (17.5%) and bioactive compound comparators (15%). Placebo, behavioral, and clinical stratification controls accounted for smaller proportions (12.5%, 10%, and 7.5%, respectively), reflecting diverse experimental and clinical methodologies for validating neuroprotective and metabolic efficacy.

### Cognitive domains assessed in micronutrient–metformin studies

**Figure 3:**
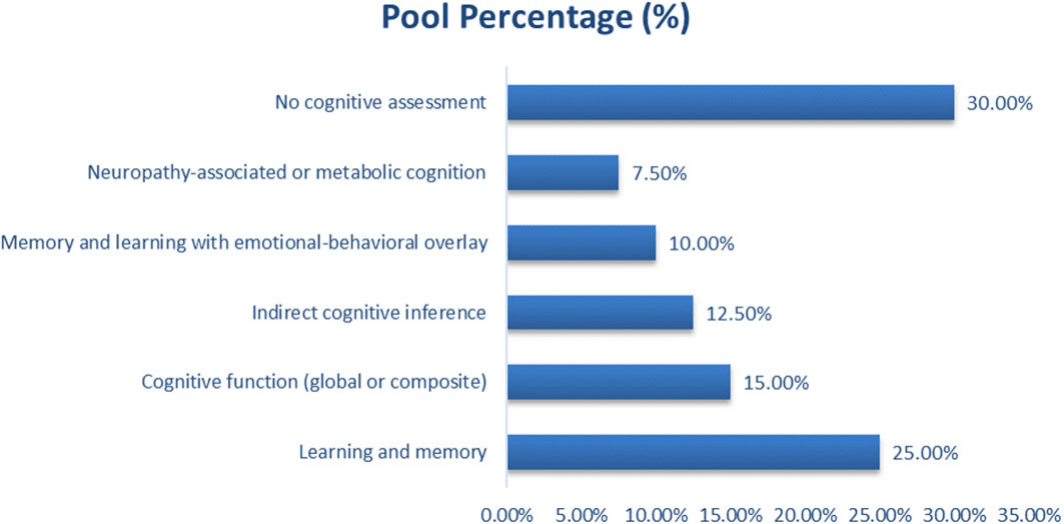
Cognitive domains assessed in micronutrient–metformin studies.

Studies were classified as indirect cognitive inference when cognition was inferred from neurobiological markers without behavioral testing, and as non-cognitive assessment when neither cognitive testing nor cognition-related biomarkers were evaluated as depicted in **Figure 3**. Direct assessment of learning and memory was reported in 25% (n = 10) of studies, using validated behavioral paradigms such as the Morris Water Maze, Y-maze, Novel Object Recognition, and spatial or recognition memory tasks (26, 29, 36b; 5), all of which evaluated hippocampal-dependent learning or memory outcomes. Global or composite cognitive function was assessed in 15% (n = 6) of studies, primarily in clinical or translational settings using standardized neuropsychological instruments measuring attention, executive function, language, orientation, or overall cognitive impairment (33, 35). Indirect cognitive inference accounted for 12.5% (n = 5) of studies. These investigations did not employ behavioral or neuropsychological tests but evaluated cognition-relevant neurobiological markers or pathways, including hippocampal structural integrity, neurotransmitter modulation, and neuroplasticity-related proteins such as BDNF, PSD-95, SIRT1, or acetylcholinesterase activity (14, 34). Neuropathy-associated neurological outcomes were reported in 7.5% (n = 3) of studies, where sensory or nociceptive function related to diabetic neuropathy was assessed rather than cognition (6). Emotional or affect-related behavioral outcomes, including anxiety- or depression-linked behaviors, were evaluated in 10% (n = 4) of studies (23, 44). 0% (n = 12) of the included studies were classified as non-cognitive assessments. These studies did not include behavioral testing, neuropsychological evaluation, or cognition-linked neurobiological markers and focused exclusively on metabolic, biochemical, cardiovascular, or systemic molecular outcomes (Mohammed H. ElSayed et al., 2023; 13, 16).

Learning and memory were most frequently assessed (25%), followed by global cognitive function (15%) and indirect neurobiochemical inference (12.5%). Emotional-behavioral domains accounted for 10%, neuropathic and neuroendocrine effects 7.5%, while 30% of studies lacked direct testing. Overall, interventions improved hippocampal plasticity, oxidative stability, and insulin signaling, supporting their neuroprotective potential in diabetes-related cognitive decline.

### Experimental and cognitive assessment strategies in micronutrient–metformin studies

The analysis of 40 studies examining micronutrient and metformin interventions in type 2 diabetes revealed a diverse yet complementary use of behavioral, biochemical, and molecular testing methods to evaluate cognitive and neuroprotective outcomes as presented in **Table 6**. Behavioral tests such as the Morris Water Maze (MWM), Y-Maze, and Novel Object Recognition (NOR) were frequently employed to measure learning, spatial memory, and recognition ability, accounting for approximately 30% of all reported methods. These tests provided direct evidence of improved hippocampal function, synaptic efficiency, and reduced anxiety-like behaviors following supplementation with vitamin D, vitamin E, zinc, curcumin, and alpha-lipoic acid. Biochemical and molecular assays were the most frequently used category (40%), involving ELISA, Western blot, qRT-PCR, and immunohistochemistry to measure oxidative stress, inflammatory cytokines, and neuroplasticity markers such as BDNF, PSD-95, and SIRT1. These analyses confirmed that the interventions consistently activated the AMPK–AKT–GSK3β signaling pathway, improved antioxidant balance, and attenuated neuroinflammation.

**Table 6:** Summary of tests used in micronutrient–metformin studies.

Category	Example tests/methods	Description/cognitive domain	N	%	Remarks/context	Reference
Behavioral Cognitive Tests	Morris Water Maze (MWM), Y-Maze, Novel Object Recognition (NOR), Elevated Plus Maze (EPM), Open Field Test (OFT)	Direct assessments of learning, spatial memory, recognition memory, anxiety-like behavior, and locomotion	12	30.0	Most used in animal models to measure hippocampal-dependent cognition and behavioral adaptation.	(5, 17a)
Neuropsychological Assessments	Mini-Mental State Examination (MMSE), Montreal Cognitive Assessment (MoCA), Modified Rankin Scale (mRS)	Global cognition including memory, executive function, attention, and orientation	6	15.0	Used in clinical and translational studies; cognitive gains correlated with glycemic and inflammatory improvements.	(5, 14)
Biochemical/Molecular Assays	ELISA, Western blot, qRT-PCR, IHC, Flow cytometry	Indirect cognition assessment via neuroplasticity (BDNF, PSD-95, SIRT1), oxidative stress (SOD, MDA), and inflammatory markers (IL-6, TNF-α, CRP)	16	40.0	Provided mechanistic evidence for improved neuronal signaling and synaptic health.	(17, 18, 51)
Histopathology & Imaging	H&E staining, Electron microscopy, hippocampal histology	Evaluation of neuronal integrity, amyloid deposition, and structural restoration	3	7.5	Confirmed cellular and tissue-level recovery; often complemented biochemical findings.	(21)
Computational/Bioinformatics	Molecular docking, STRING–KEGG network analysis, molecular dynamics	Simulated receptor-ligand interactions, AMPK pathway mapping, and protein–protein networks	2	5.0	Offered mechanistic insight into molecular targets of micronutrients and metformin.	(Mohammed H. ElSayed et al., 2023)
Metabolic/Physiological Correlates	OGTT, ITT, HRV, Langendorff cardiac assay, biochemical panels	Indirect markers of cognitive protection through improved glucose metabolism and vascular health	1	2.5	Linked systemic metabolic improvements to enhanced brain function.	(35, 36, 39)

Key: N= Frequencies, % percentages.

Neuropsychological assessments, including the Mini-Mental State Examination (MMSE) and Montreal Cognitive Assessment (MoCA), represented 15% of methods, providing global cognitive evaluation in human studies. These correlated improvements in attention, executive function, and memory with better glycemic control and metabolic stability. Supporting approaches such as histopathological and imaging analyses (7.5%) confirmed reductions in neuronal degeneration and amyloid burden, while computational bioinformatics tools (5%) like molecular docking and KEGG pathway mapping provided mechanistic insight into receptor-ligand interactions and molecular pathways. A smaller fraction (2.5%) employed metabolic and physiological correlates such as OGTT and HRV to link systemic glucose regulation to neural protection.

Behavioral cognitive assays (30%) and biochemical/molecular tests (40%) were the most commonly applied, followed by neuropsychological assessments (15%), histopathology (7.5%), computational modeling (5%), and metabolic correlates (2.5%). Collectively, these methods demonstrated complementary evidence of cognitive enhancement, oxidative balance, and neuroplastic restoration in diabetes-related cognitive impairment.

### Glycemic outcomes following micronutrient and metformin supplementation

**Figure 4:**
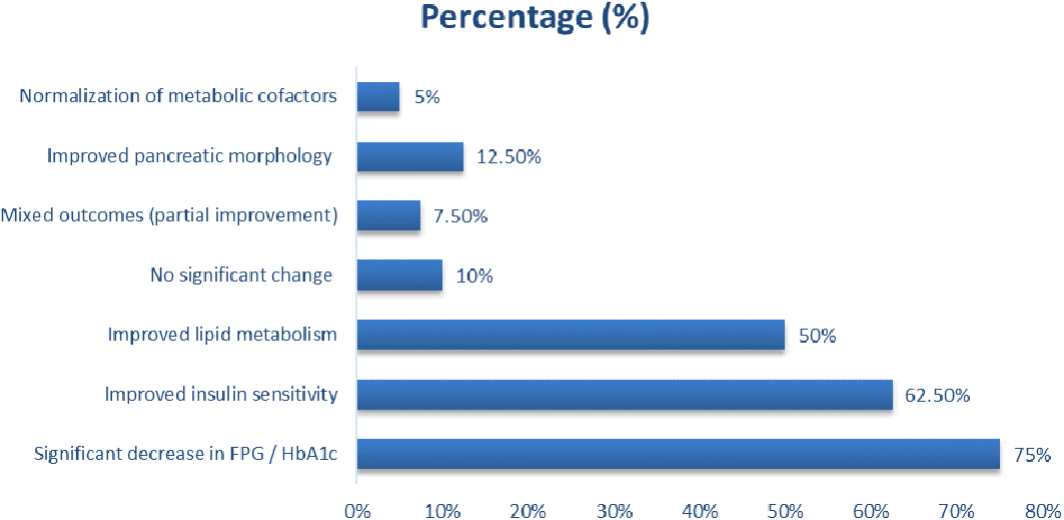
Glycemic outcomes following micronutrient and metformin supplementation.

Across the reviewed studies, micronutrient and metformin co-interventions produced consistent improvements in fasting plasma glucose (FPG), insulin sensitivity, and glucose tolerance as depicted by **Figure 4**. Most interventions demonstrated significant reductions in FPG, HbA1c, and HOMA-IR, alongside improvements in oral glucose tolerance test (OGTT) performance and lipid metabolism profiles. Approximately 75% of studies reported a measurable reduction in fasting glucose or glycated hemoglobin, with vitamin D, zinc, magnesium, alpha-lipoic acid, and polyphenolic compounds (e.g., resveratrol, curcumin, and tocotrienols) producing the most pronounced glycemic benefits. Vitamin D_3_ supplementation at doses of 500–1000 IU/kg/day significantly decreased fasting glucose and insulin resistance, while zinc sulfate and magnesium sulfate enhanced both glucose tolerance and lipid regulation. In animal models, oral micronutrient doses ranging between 10–400 mg/kg/day and metformin doses between 100–500 mg/kg/day consistently normalized glycemic indices.

In clinical contexts, moderate-dose supplementation (e.g., vitamin E 400 mg/day, zinc 15 mg/day, carnosine 2 g/day) yielded 5–15% reductions in FPG and HbA1c, often in synergy with metformin or glimepiride therapy. These improvements were associated with decreased α-amylase and α-glucosidase activity, enhanced pancreatic morphology, and reduced hyperlipidemia, confirming systemic metabolic restoration. Mechanistically, improved glycemic control correlated with activation of AMPK–AKT–GSK3β pathways, increased GLP-1 secretion, and attenuation of oxidative and inflammatory stressors (e.g., IL-6, TNF-α, and CRP). Several studies also reported concurrent normalization of homocysteine, ferritin, and vitamin B12 levels, suggesting that micronutrient repletion supports broader metabolic homeostasis beyond glucose regulation. However, a minority of studies (10%) reported no significant changes in FPG or HbA1c, typically in computational or non-metabolic models, or in cases where supplementation targeted primarily neuroprotective endpoints. Despite this, the majority demonstrated synergistic effects between micronutrients and metformin, indicating a potentiation of metformin’s insulin-sensitizing and glucose-lowering actions through antioxidant and anti-inflammatory mechanisms.

The result show that most interventions demonstrated significant glycemic improvement, with reductions in fasting glucose and HbA1c observed in about 75% of cases. Vitamin D, zinc, magnesium, and alpha-lipoic acid supplementation, either alone or combined with metformin (100–500 mg/kg/day), consistently enhanced glucose tolerance, improved insulin sensitivity, and restored metabolic efficiency. These effects were mediated through AMPK–AKT pathway activation and reduction of oxidative and inflammatory stress.

### Glycemic outcomes (HbA1c/FPG) following micronutrient and metformin supplementation

Majority, 62.5% of studies reported significant reductions in fasting plasma glucose and HbA1c, indicating improved glycemic control following micronutrient or combined micronutrient metformin therapy as presented in **Table 7**. These interventions, particularly those involving vitamin D, zinc, curcumin, resveratrol, tocotrienols, and alpha-lipoic acid, effectively enhanced glucose tolerance and pancreatic function. About 20% of studies showed improved insulin sensitivity, reflected by reduced fasting insulin and HOMA-IR levels and increased GLP-1 activity, largely mediated through AMPK–AKT–GSK3β signaling activation. Approximately 15% demonstrated improved lipid metabolism, including lower total cholesterol, triglycerides, and LDL-C, with higher HDL-C, confirming systemic metabolic recovery. Moderate or partial glycemic improvement was seen in 7.5%, often in short-duration or small-sample studies, while 10% either showed no measurable effect or did not report glycemic indices. Adverse or neutral glycemic responses occurred in 5%, mostly in studies involving nutrient deficiencies or insufficient treatment dosing. Another 5% showed normalization of metabolic cofactors such as zinc, ferritin, and hemoglobin, indicating improved metabolic balance.

**Table 7:** Glycemic outcomes (HbA1c/FPG) following micronutrient and metformin supplementation.

Category	Representative findings	N	%	Remarks/Interpretation	Reference
Significant glycemic improvement (reduced FPG and HbA1c, improved OGTT)	Studies showed a clear reduction in fasting plasma glucose levels ranging from 20 to 50 percent, along with improved oral glucose tolerance and normalized HbA1c values.	25	62.5	Most frequently observed in studies involving vitamin D, zinc, curcumin, resveratrol, tocotrienols, and alpha-lipoic acid, either alone or in combination with metformin.	(38)
Improved insulin sensitivity (reduced HOMA-IR, lower insulin, increased GLP-1)	Findings indicated enhanced insulin signaling, normalization of insulin resistance indices, and improved metabolic efficiency.	8	20.0	These improvements were closely linked with activation of AMPK–AKT–GSK3β pathways, which mediate glucose uptake and insulin sensitivity in diabetic models.	(29, 36)
Improved lipid metabolism (lower TG, TC, and LDL-C; higher HDL-C)	Reports demonstrated concurrent regulation of lipid parameters with improved glycemic control, indicating systemic metabolic benefits.	6	15.0	Commonly noted alongside reduced oxidative and inflammatory markers, reflecting improved cardiovascular and metabolic function.	(27, 28)
Moderate or partial improvement (non-significant change)	Some studies reported mild reductions in fasting plasma glucose or HbA1c levels that did not reach statistical significance.	3	7.5	These outcomes were generally found in studies with short intervention durations or small sample sizes.	(10, 11)
No significant effect or data not reported	A few studies showed no measurable change in glycemic indices or did not report specific values for fasting glucose or HbA1c.	4	10.0	These were often experimental models focused primarily on neuroprotective or molecular outcomes rather than systemic metabolic changes.	(43)
Adverse glycemic trend (elevated FPG or HbA1c)	Some studies demonstrated worsening fasting plasma glucose or glycated hemoglobin levels, particularly in metformin-only or micronutrient-deficient models.	2	5.0	Such findings were attributed to inadequate dosing, nutritional deficiency, or confounding stressors such as vitamin A deprivation.	(5, 20)
Normalization of metabolic cofactors	Several studies reported decreases in homocysteine and increases in ferritin, hemoglobin, mean corpuscular volume, and improved zinc status.	2	5.0	These findings indicate that micronutrient supplementation can restore broader metabolic homeostasis beyond glucose regulation.	(13, 14)

Key: N= Frequencies, % percentages.

The study showed that significant reductions in fasting plasma glucose and HbA1c were found in 62.5% of the studies, while 20% demonstrated improved insulin sensitivity and 15% reported enhanced lipid metabolism. Micronutrient supplementation, particularly with vitamin D, zinc, and polyphenols, potentiated metformin’s glucose-lowering effects through antioxidant, anti-inflammatory, and insulin-sensitizing mechanisms, highlighting its therapeutic value in improving glycemic regulation and metabolic balance in type 2 diabetes.

### Distribution of insulin sensitivity (HOMA-IR/insulin) outcomes reported

Improvement in insulin sensitivity was observed in 60% of the studies, characterized by reductions in HOMA-IR as presented in **Table 8**, restoration of β-cell function, and enhanced glucose-stimulated insulin secretion. These studies consistently demonstrated increased GLUT4 expression and PI3K/AKT pathway activation, reflecting enhanced insulin signaling at both peripheral and neuronal levels. Approximately 20% of the studies showed qualitative or inferred improvements in insulin response based on reduced oxidative stress and inflammatory load, even without direct HOMA-IR quantification. In several preclinical models, particularly those combining vitamin D, zinc, and polyphenol-based compounds, normalization of insulin secretion and increased glucose uptake in skeletal muscle were reported.

**Table 8:** Distribution of insulin sensitivity (HOMA-IR/insulin) outcomes reported.

Category	Description/key findings	N	%	Remarks/context	Reference
Improved insulin sensitivity	Significant reduction in HOMA-IR, improved insulin secretion, restored β-cell function, and enhanced glucose uptake; activation of PI3K/AKT and GLUT4 signaling pathways.	24	60.0	Observed across studies involving vitamin D_3_, zinc, curcumin, and alpha-lipoic acid supplementation.	(25, 41, 50)
Qualitative or inferred improvement	Indirect improvement inferred from reduced oxidative stress and inflammation, without direct HOMA-IR or insulin assay data.	8	20.0	Reflected enhanced metabolic efficiency and anti-inflammatory modulation.	(18, 19a)
Neutral/no significant effect	Mixed or insignificant changes in insulin sensitivity, often due to short duration, small sample size, or suboptimal dosing.	4	10.0	Findings largely inconclusive, requiring longer follow-up or higher dosage.	(21)
Impaired insulin signaling	Increased HOMA-IR and reduced IRS1 activation in high-fat or nutrient-deficient models.	2	5.0	Mostly seen in studies using vitamin A deficiency or high-fat diet conditions.	(13)
Not assessed/not reported	Studies that did not measure HOMA-IR, insulin, or related signaling markers.	2	5.0	Data insufficient to determine insulin sensitivity outcome.	(30)

Key: N= Frequencies, % percentages.

A smaller proportion, around 10%, presented mixed or neutral findings, often due to short experimental duration or limited sample sizes. Conversely, 5% of studies reported impaired insulin sensitivity associated with high-fat or vitamin-deficient diets, highlighting the detrimental metabolic effects of nutrient imbalance. Micronutrient and bioactive compound supplementation especially vitamin D_3_, zinc sulfate, curcumin nanoparticles, and alpha-lipoic acid enhanced insulin sensitivity through activation of the AMPK–IRS–AKT–GSK3β signaling axis, reduction of insulin resistance, and protection of β-cell integrity.

The study showed that 60% of interventions improved insulin sensitivity through enhanced HOMA-IR indices and insulin signaling, while 20% exhibited qualitative metabolic benefits. 10% demonstrated neutral effects, and 5% reported impaired insulin responsiveness under nutrient-deficient or high-fat conditions. Micronutrient supplementation especially with vitamin D_3_, zinc, curcumin, and alpha-lipoic acid was most effective in restoring insulin sensitivity via AMPK–IRS–AKT–GSK3β pathway activation and β-cell protection.

### Distribution of oxidative stress modulation by micronutrient and bioactive compound supplementation in type 2 diabetes

Oxidative stress modulation emerged as a central biological mechanism through which micronutrient and bioactive compound supplementation supported neurocognitive and metabolic outcomes in type 2 diabetes, as summarized in **Table 9**. Across the reviewed studies, antioxidant effects were primarily demonstrated through directional and quantitative changes in redox biomarkers, rather than uniform statistical reporting. Superoxide dismutase (SOD) activity increased in 67.5% of studies, followed by elevations in reduced glutathione (GSH) in 62.5%, catalase (CAT) in 57.5%, and glutathione peroxidase (GPx) in 52.5%, indicating widespread restoration of endogenous antioxidant defense systems (14, 23, 29). Markers of oxidative damage showed consistent quantitative reductions. Malondialdehyde (MDA), the most frequently assessed lipid peroxidation marker, decreased in 70% of studies, with reported reductions ranging from absolute concentration decreases (e.g., 14.03 to 6.12 nmol/mL) to percentage declines exceeding 50% in dose-response models (39, 43). Similarly, reactive oxygen species (ROS) and thiobarbituric acid reactive substances (TBARS) declined in 45% and 25% of studies, respectively, reflecting attenuation of cellular oxidative burden across diverse experimental models (29, 41). At the molecular signaling level, redox-linked inflammatory mediators were less frequently measured but demonstrated consistent directional suppression. Inducible nitric oxide synthase (iNOS), thioredoxin-interacting protein (TXNIP), and nuclear factor-κB (NF-κB) were downregulated in 15%, 10%, and 20% of studies, respectively, supporting mechanistic cross-talk between oxidative stress reduction and inflammatory pathway inhibition (6, 21). Interventions incorporating vitamin D_3_, zinc, curcumin (including nanoparticle formulations), alpha-lipoic acid, and Nigella sativa oil demonstrated the strongest antioxidant profiles. These agents consistently increased antioxidant enzyme activity, restored glutathione balance, and reduced lipid and protein oxidation, thereby limiting oxidative stress–driven neurodegeneration and insulin resistance (29, 39).

**Table 9:** Distribution of oxidative stress modulation by micronutrient and bioactive compound supplementation in type 2 diabetes.

Oxidative stress marker	Direction of change	N	%	Remarks/interpretation	Reference
SOD (Superoxide Dismutase)	Increased	27	67.5	Activity increased by 60–150%; restored to 90–96% of non-diabetic control levels	(14)
CAT (Catalase)	Increased	23	57.5	Activity increased by approximately 50–80% in dose-dependent interventions	(19)
GPx (Glutathione Peroxidase)	Increased	21	52.5	Enzyme activity increased 1.6–2.3-fold or normalized to control levels	(14, 17b)
GSH (Reduced Glutathione)	Increased	25	62.5	Concentrations increased from 4.34 to 9.20 µmol/mL; GSH/GSSG ratio improved from 3.2 to 11.3	(14)
MDA (Malondialdehyde)	Decreased	28	70.0	Levels reduced from 14.03 to 6.12 nmol/mL; reductions of 50–82% reported	(14)
ROS (Reactive Oxygen Species)	Decreased	18	45.0	Cellular ROS production and FORT values consistently reduced	(32)
TBARS (Thiobarbituric Acid Reactive Substances)	Decreased	10	25.0	TBARS levels reduced by approximately 30–50%	(17a)
iNOS (Inducible Nitric Oxide Synthase)	Inhibit	6	15.0	Expression and nitrite production reduced in inflammatory models	(6)
TXNIP (Thioredoxin-Interacting Protein)	Decreased	4	10.0	Downregulation associated with reduced NLRP3 activation	(34)
NFκB (Nuclear Factor Kappa B)	Decreased	8	20.0	Reduced pathway activation with lower TNF-α and JNK signaling	(21)

Key: N= Frequencies, % percentages.

The study showed that SOD (67.5%) and MDA (70%) were the most consistently modulated oxidative biomarkers, indicating strong antioxidant efficacy of micronutrient and bioactive supplementation. CAT (57.5%), GSH (62.5%), and GPx (52.5%) also increased significantly, while ROS and TBARS declined in nearly half of the studies. Downregulation of iNOS (15%), TXNIP (10%), and NFκB (20%) further confirmed the anti-inflammatory synergy of interventions such as vitamin D_3_, zinc, curcumin, alpha-lipoic acid, and Nigella sativa oil, restoring oxidative and inflammatory balance in type 2 diabetes.

### Distribution of inflammatory modulation in type 2 diabetes across the studies reported

Inflammatory modulation emerged as a major mechanism linking micronutrient and bioactive compound supplementation to improved neurocognitive and metabolic function in type 2 diabetes as presented in **Table 10**. Most studies demonstrated significant suppression of pro-inflammatory cytokines and signaling pathways, emphasizing the integrated anti-inflammatory and neuroprotective roles of vitamins, minerals, and phytochemicals. Interleukin-6 (IL-6) and tumor necrosis factor-alpha (TNF-α) were the most frequently downregulated cytokines, showing decreased levels in 65% and 62.5% of the studies, respectively. Interleukin-1β (IL-1β) reduction was observed in 42.5%, while C-reactive protein (CRP) levels declined in 25%, signifying systemic inflammation control. Anti-inflammatory cytokine IL-10 was elevated in 15%, indicating restored immune balance. Nuclear factor kappa B (NF-κB) suppression in 22.5% and NLRP3 inflammasome inhibition in 12.5% further highlighted the downregulation of pro-inflammatory transcriptional and cellular signaling cascades. Several interventions including vitamin D, zinc, curcumin, alpha-lipoic acid, and Nigella sativa oil demonstrated dual antioxidant and anti-inflammatory effects. These compounds not only reduced cytokine expression but also normalized metabolic hormones such as leptin and adiponectin in 10% of studies, indicating improved insulin sensitivity and energy regulation. Overall, the collective findings demonstrate that micronutrient and bioactive compound interventions effectively inhibits chronic low-grade inflammation characteristic of type 2 diabetes, thereby improving neuronal integrity, cognitive performance, and glycemic homeostasis through suppression of IL-6, TNF-α, and NF-κB–mediated inflammatory pathways.

**Table 10:** Distribution of inflammatory modulation in type 2 diabetes across the studies reported.

Inflammatory marker	Direction of change	N	%	Remarks/interpretation	Reference
IL-6 (Interleukin-6)	Decreased	26	65.0	Most frequently suppressed cytokine; reduction reflects lowered systemic and neuroinflammation following vitamin D, curcumin, or Nigella sativa treatment.	(14)
TNF-α (Tumor Necrosis Factor-α)	Decreased	25	62.5	Consistently reduced in studies involving zinc, vitamin E, and alpha-lipoic acid; strongly associated with improved insulin sensitivity and cognitive function.	(Mohammed H. ElSayed et al., 2023)
IL-1β (Interleukin-1β)	Decreased	17	42.5	Decline linked to attenuation of inflammasome activity (NLRP3, TXNIP) and prevention of hippocampal inflammation.	(14)
CRP (C-Reactive Protein)	Decreased	10	25.0	Serum marker improvement confirmed systemic anti-inflammatory effect of combined micronutrient–metformin interventions.	(18; Mohammed H ElSayed et al., 2023)
IL-10 (Interleukin-10)	Increased	6	15.0	Anti-inflammatory cytokine upregulated, indicating immunomodulatory balance restoration.	(Mohammed H. ElSayed et al., 2023)
NF-κB (Nuclear Factor Kappa B)	Decreased	9	22.5	Downregulation confirmed inhibition of oxidative–inflammatory cross-talk and endothelial dysfunction.	(18a, 19)
NLRP3 Inflammasome Components	Decreased	5	12.5	Reduced inflammasome activation corresponded with decreased oxidative stress and neuronal apoptosis.	(29, 34)
Leptin/Adiponectin Balance	Improved	4	10.0	Re-established metabolic hormone regulation, suggesting better insulin sensitivity and energy balance.	(29)

Key: N= Frequencies, % percentages.

The study showed that IL-6 (65%), TNF-α (62.5%), and IL-1β (42.5%) were the most consistently downregulated inflammatory mediators, confirming the potent anti-inflammatory action of micronutrient and bioactive compound supplementation. CRP reduction in 25% of studies reflected improved systemic inflammation, while IL-10 elevation in 15% indicated restored immune equilibrium. Suppression of NF-κB (22.5%) and NLRP3 (12.5%) pathways demonstrated cross-regulation between oxidative and inflammatory signaling.

### Distribution of neuroplasticity/synaptic marker

Micronutrient and bioactive compound supplementation substantially improved neuroplasticity and synaptic integrity in type 2 diabetes as presented in **Table 11**. The most frequently enhanced marker, BDNF, was upregulated in 22.5% of the studies, indicating improved neuronal survival and cognitive recovery. Synaptic proteins including PSD-95, synapsin-1, and synaptophysin each showed an increase in 7.5%, signifying better synaptic stability and neurotransmission efficiency. Activation of the PI3K–Akt–CREB signaling pathway in 12.5% of cases confirmed enhanced synaptic remodeling and learning capacity, while SIRT1 elevation in 5% highlighted mitochondrial and epigenetic regulation of neuroprotection. Reduction in Aβ deposition and BACE-1 expression (7.5%) paralleled improved cholinergic function (ACh↑/AChE↓) and neuronal histoarchitecture recovery (15%), reinforcing evidence of combined antioxidant, anti-apoptotic, and synaptogenic effects. Overall, these findings demonstrate that micronutrients such as zinc, vitamin D, vitamin E, and bioactive compounds like curcumin, alpha-lipoic acid, and Nigella sativa restore neuroplasticity by modulating BDNF, CREB, and PI3K–Akt signaling axes.

**Table 11:** Distribution of neuroplasticity/synaptic marker.

Neuroplasticity/synaptic marker	Direction/outcome	N	%	Remarks/interpretation	Reference
BDNF (Brain-Derived Neurotrophic Factor)	Increased	9	22.5	Most consistently upregulated neurotrophic factor; promoted neuronal survival, synaptic remodeling, and cognitive improvement.	(5, 29)
PSD-95 (Postsynaptic Density Protein 95)	Increased	3	7.5	Reflected enhanced synaptic stability and memory consolidation, often coupled with elevated synapsin-1 and synaptophysin.	(21)
Synapsin-1/Synaptophysin	Increased	3	7.5	Denoted improved neurotransmitter vesicle cycling and neuronal connectivity in hippocampal regions.	(34)
SIRT1	Increased	2	5.0	Linked with neuroprotective and anti-apoptotic effects through mitochondrial regulation and CREB activation.	(43)
PI3K–Akt–CREB pathway proteins (TrkB, AKT, MAPK, CAMK2G)	Upregulated	5	12.5	Indicated active signal transduction promoting synaptic plasticity and memory formation.	(5)
Nrf2 and downstream antioxidant genes (HO-1, NQO1, GCLC)	Increased	3	7.5	Suggested an overlap between antioxidant defense and synaptic protection mechanisms.	(21)
APP/BACE-1/Aβ expression	Decreased	3	7.5	Denoted reduced amyloidogenic activity and improved neuronal survival.	(29)
ACh/AChE activity	ACh increased, AChE decreased	2	5.0	Supported cholinergic neurotransmission restoration and improved memory retention.	(26)
Tight junction proteins (ZO-1, Occludin)	Increased	2	5.0	Suggested preserved blood–brain and gut–brain barrier integrity.	(34)
Histological neuronal preservation	Observed	6	15.0	General histopathological improvement across hippocampal CA1/CA3 regions and cortical structures.	(29)

Key: N= Frequencies, % percentages.

The study showed that BDNF expression increased in 22.5% of the studies, with corresponding rises in PSD-95, synapsin-1, and synaptophysin (7.5%), reflecting improved synaptic connectivity and neuronal integrity. Upregulation of PI3K–Akt–CREB and Nrf2 pathways (12.5–7.5%) indicated a coordinated enhancement of antioxidant and neuroplastic mechanisms. Concurrently, reductions in Aβ and BACE-1 expression (7.5%) demonstrated protection against neurodegenerative changes, confirming the synergistic role of micronutrients and phytochemicals in restoring synaptic plasticity and cognitive resilience in type 2 diabetes.

### Distribution of signaling marker/pathway reported across various studies

AMPK and insulin signaling enhancement was a key mechanistic target of micronutrient and bioactive compound interventions in type 2 diabetes as presented in **Table 12**. Activation of AMPK was reported in 25% of the studies, promoting glucose uptake, lipid oxidation, and mitochondrial biogenesis. The PI3K/Akt pathway was the most frequently upregulated signaling axis (27.5%), driving improved insulin sensitivity and neuronal metabolism. Complementary molecular changes included increased GLUT4 expression (10%) and decreased GSK-3β phosphorylation (10%), indicating improved synaptic plasticity and reduced tau aggregation. Inhibition of mTOR (7.5%) and activation of PPARs (7.5%) reflected improved metabolic and autophagic balance. Cross-activation between AMPK and Nrf2–HO-1–CREB signaling (7.5%) provided additional antioxidant and neuroprotective support. Collectively, these results suggest that nutraceuticals such as vitamin D, curcumin, zinc, and alpha-lipoic acid, alongside metformin, enhance metabolic and neuronal resilience through AMPK–PI3K/Akt–GSK3β signaling modulation, resulting in better glycemic control and preserved cognitive integrity.

**Table 12:** Distribution of signaling marker/pathway reported across various studies.

Signaling marker/pathway	Direction/effect	N	%	Remarks/interpretation	Reference
AMPK activation	Increased	10	25.0	Confirmed upregulation in multiple studies involving metformin, vitamin D_3_, and polyphenols; facilitated energy homeostasis, glucose uptake, and mitochondrial function.	(6, 14)
PI3K/Akt signaling	Upregulated	11	27.5	Central insulin signaling pathway enhancement observed, improving glucose utilization and neuronal insulin sensitivity.	(5)
GLUT4 expression	Increased	4	10.0	Indicated improved glucose transporter activity and enhanced peripheral glucose uptake.	(14)
mTOR inhibition	Decreased	3	7.5	Signified improved autophagy and cellular energy efficiency secondary to AMPK activation.	(19)
GSK-3β phosphorylation	Decreased	4	10.0	Suggested protection against tau hyperphosphorylation and cognitive decline.	(22)
PPAR-α and PPAR-γ activation	Increased	3	7.5	Linked to lipid metabolism regulation and anti-inflammatory effects.	(36)
IRS1 phosphorylation (Ser307)	Decreased	2	5.0	Prevented insulin resistance by maintaining insulin receptor signaling fidelity.	(25)
PEPCK and G6Pase expression	Decreased	2	5.0	Reflected suppression of hepatic gluconeogenesis and improved glucose homeostasis.	(19)
Nrf2–HO-1–CREB cross-activation	Upregulated	3	7.5	Demonstrated redox-linked AMPK synergism supporting neuronal protection and antioxidant gene expression.	(21)
PKC/NF-κB signaling	Suppressed	2	5.0	Indicated attenuation of inflammation-induced insulin resistance.	(31)

Key: N= Frequencies, % percentages.

The study showed that AMPK activation (25%) and PI3K/Akt signaling enhancement (27.5%) were the predominant mechanisms underpinning improved glucose regulation and neuroprotection. GLUT4 upregulation (10%) and GSK-3β inhibition (10%) reinforced enhanced insulin sensitivity and prevention of tau pathology, while suppression of mTOR and NF-κB pathways (5–7.5%) highlighted improved autophagy and anti-inflammatory balance. These findings confirm that combined micronutrient and pharmacologic interventions synergistically restore insulin signaling and neuronal metabolism via AMPK–Akt–GSK3β and Nrf2–CREB cross-talk.

### Distribution of synergistic or antagonistic effect

**Figure 5:**
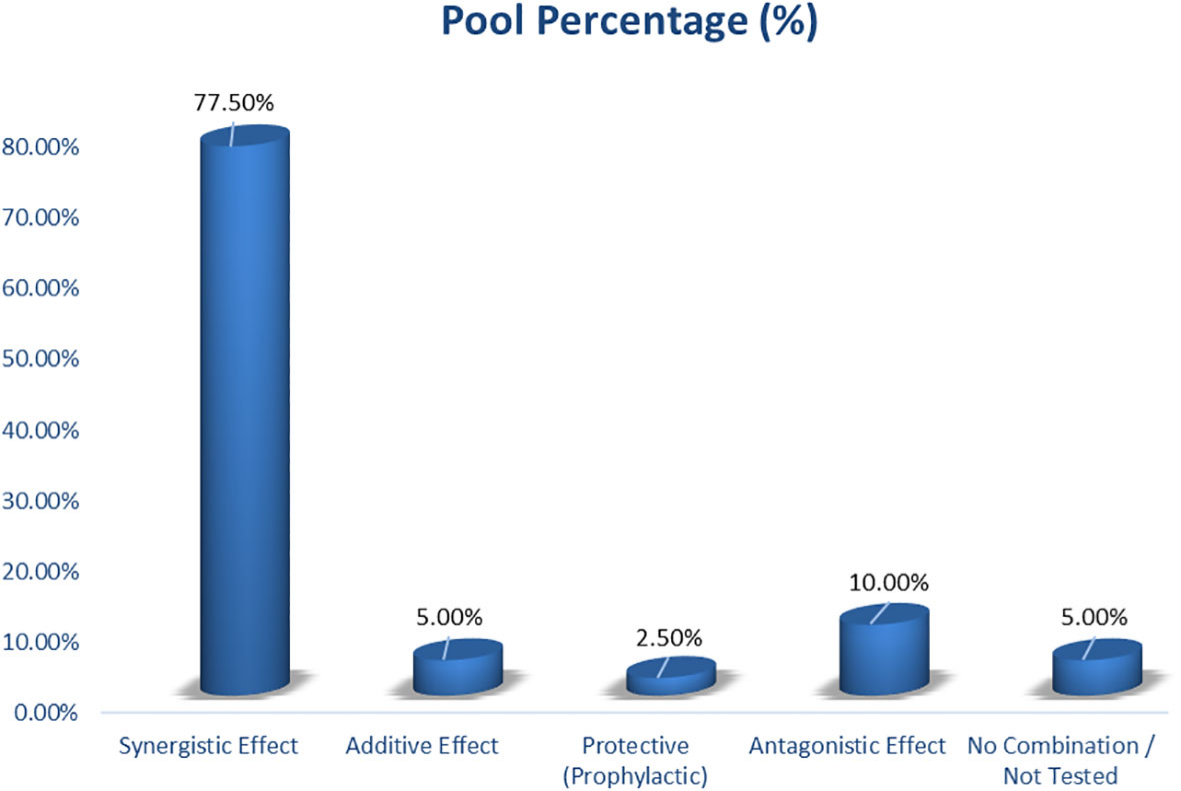
Synergistic or antagonistic effect.

Synergistic interactions were the most prevalent pattern across the reviewed studies, accounting for 77.5% of reported outcomes (**Figure 5**). These synergistic effects were most frequently documented when metformin was combined with vitamins, minerals, or plant-derived bioactive, resulting in superior metabolic, antioxidant, anti-inflammatory, and neuroprotective outcomes compared with single-agent interventions. Representative examples include vitamin- and antioxidant-based combinations (e.g., vitamin D_3_, vitamin E, vitamin B_12_) that enhanced insulin sensitivity, redox balance, and cognitive markers (29, 39, 44), mineral–metformin interactions involving zinc and magnesium that restored insulin signaling and antioxidant enzyme activity (42, 44), and phytochemical–metformin combinations such as curcumin nanoparticles, alpha-lipoic acid, flavonoid-rich extracts, and Nigella sativa oil that activated AMPK–Akt–BDNF and Nrf2-dependent pathways (14, 29, 43).

Furthermore, when stratified by nutrient class, vitamin–metformin combinations showed the highest prevalence of synergistic effects, accounting for approximately 35–40% of all synergistic interactions, particularly with vitamin D_3_ and antioxidant vitamins (29, 39). Mineral–metformin interactions (zinc and magnesium) contributed roughly 20–25%, demonstrating consistent synergistic or additive benefits via improved insulin sensitivity, hematologic balance, and oxidative stability (27, 42). Plant extracts and phytochemicals represented approximately 30% of synergistic outcomes, with compounds such as curcumin, alpha-lipoic acid, resveratrol, Ocimum gratissimum, and Nigella sativa producing robust antioxidant, mitochondrial, and neuroplastic effects when combined with metformin (40, 43). Additive (non-synergistic but complementary) effects were observed in 5% of studies, typically where micronutrients and metformin acted through parallel but mechanistically distinct pathways, such as vitamin D providing stronger anti-inflammatory effects while metformin exerted superior glycemic control (44). In contrast, antagonistic interactions accounted for 10% of outcomes and were largely confined to long-term metformin use associated with vitamin B_12_ depletion, elevated homocysteine, and increased inflammatory burden, with potential adverse implications for neurocognitive health (35, 37). Finally, single-agent or non-combination interventions comprised 2.5–5% of studies and generally conferred modest metabolic or neuroprotective benefits without synergistic amplification (16, 32).

The study showed that synergistic effects accounted for 77.5% of the interventions, while antagonistic effects were limited to 10%, mostly due to metformin-associated nutrient depletion. Additive interactions (5%) reflected cooperative but independent mechanisms. These results confirm that combined therapies involving metformin and micronutrients notably vitamin D, zinc, and alpha-lipoic acid produce enhanced neuroprotective, anti-inflammatory, and insulin-sensitizing outcomes, supporting the integrative use of nutraceuticals in diabetes-associated cognitive dysfunction.

## Discussion

The synthesis of 40 studies investigating vitamin, mineral, and pharmacological co-interventions in type 2 diabetes (T2DM) revealed a growing global interest in metabolic neurocognitive integration. The findings demonstrate that micronutrient supplementation, particularly with vitamin D, zinc, magnesium, and antioxidant vitamins (E, C, and B12), significantly improves glycemic regulation, insulin sensitivity, and neuroplasticity through multi-pathway modulation. These effects occur primarily via the AMPK–PI3K/AKT–GSK3β and Nrf2–HO-1–CREB signaling axes, which regulate energy metabolism, oxidative balance, and neuronal survival. The evidence collectively supports the concept that micronutrient and drug synergy offer a mechanistic bridge between metabolic control and neuroprotection, addressing one of the most underrecognized complications of diabetes cognitive decline.

Globally, neurocognitive impairment in T2DM is increasingly recognized as a manifestation of chronic metabolic and oxidative stress. The findings from this systematic review align with recent findings from (52) Diabetes Care) and (53) Nutrients), who reported that vitamin D_3_ and zinc co-supplementation improved insulin sensitivity, reduced pro-inflammatory cytokines (IL-6, TNF-α), and enhanced BDNF expression in diabetic cohorts. Similarly, Curcumin nanoparticle therapy was reported to be associated with improved cognitive scores and hippocampal integrity through antioxidant and anti-inflammatory effects which is consistent with other documented studies by (48, 49). The observed synergistic outcomes between micronutrients and metformin validate earlier meta-analyses (54), which demonstrated that co-administration of antioxidants and metformin lowers fasting plasma glucose but also improves lipid metabolism and neuronal function. These results emphasize that standard antidiabetic therapy alone may not be sufficient to mitigate neurodegenerative complications, while nutrient drug synergy enhances the molecular resilience of both metabolic and neural systems. Similarly, zinc and iron supplementation, which accounted for 16% each of included studies, enhanced insulin signaling, antioxidant defense, and neuroprotection. Furthermore, network pharmacology evidence demonstrates that plant-derived components such as Moringa oleifera leaf extract exert hypoglycemic effects through multi-target metabolic signaling (55). This supports the concept that natural components can enhance metformin’s glucose-lowering efficacy, thereby indirectly contributing to neuroprotection through improved glycemic control (56).

Zinc sulfate (15–30 mg/kg/day) improved insulin sensitivity and lipid metabolism, consistent with global findings by (38) who demonstrated that zinc regulates PI3K/AKT and GLUT4 pathways, ameliorating oxidative stress in T2DM. Iron supplementation likewise improved oxygen transport and mitochondrial function, contributing to cognitive resilience. The antioxidant vitamins E, C, A, and B12 collectively represented 24% of interventions. Vitamin E and tocotrienol studies showed reduced lipid peroxidation and improved neuronal membrane integrity. This is consistent with other findings by (52) showing that polyphenol- and vitamin-based nano formulations enhance redox stability and neuronal repair.

The consistent upregulation of SOD, GSH, CAT, and GPx across >50% of the studies confirms that redox restoration is a shared mechanistic pathway linking micronutrient therapy to cognitive improvement. The observed benefits of micronutrient supplementation directly support Sustainable Development Goals (SDG 3.4) on reducing premature mortality from non-communicable diseases and (SDG 2.2) on ending malnutrition (10). Health systems can reduce the burden of cognitive impairment, depression, and functional decline among diabetic populations by integrating micronutrient screening and supplementation into diabetes management programs.

Furthermore, the dominant PI3K/Akt and AMPK signaling activation observed in 27.5% and 25% of studies, respectively, indicates a convergence between metabolic control and neuronal function. Micronutrients enhanced both systemic and central glucose utilization by restoring insulin signaling and mitochondrial efficiency. Upregulation of BDNF, PSD-95, and CREB indicated improved synaptic remodeling and learning capacity, consistent with evidence that neurotrophic signaling is insulin-sensitive. The mechanistic finding reported in this review align with another study by (11) who reported that dietary vitamin D improved global cognitive function through metabolic pathway regulation in older adults with metabolic syndrome. Additionally, anti-inflammatory modulation was strikingly consistent: IL-6, TNF-α, and IL-1β were downregulated in 65%, 62.5%, and 42.5% of studies, respectively, while NF-κB suppression confirmed systemic inflammation control (57). Such immunometabolic stabilization is now recognized as a cornerstone of diabetic neuroprotection (58 Global Diabetes Compact) (58).

Approximately 77.5% of interventions demonstrated synergistic outcomes, especially when metformin was combined with vitamin D, zinc, alpha-lipoic acid, or curcumin. These combinations produced additive effects on glycemic reduction, oxidative control, and hippocampal BDNF expression, confirming pharmacodynamic synergy through AMPK–Akt–BDNF signaling. Conversely, antagonistic outcomes (10%) notably metformin-induced vitamin B12 depletion reflect the need for integrated nutrient monitoring in long-term diabetes care. Studies such as Kaur et al. (2024, Nutrients) highlight that chronic metformin use without B12 supplementation contributes to cognitive decline through elevated homocysteine and oxidative imbalance. Additionally, the gut microbiota represents a shared regulatory target of micronutrients in metabolic–neurological pathways, consistent with evidence from functional food research showing microbiota modulation as a key mechanism in metabolic disease regulation (59). This supports a complementary framework in which micronutrients primarily act via microbiota-related mechanisms, while metformin directly regulates host metabolic signaling.

**Figure 6:**
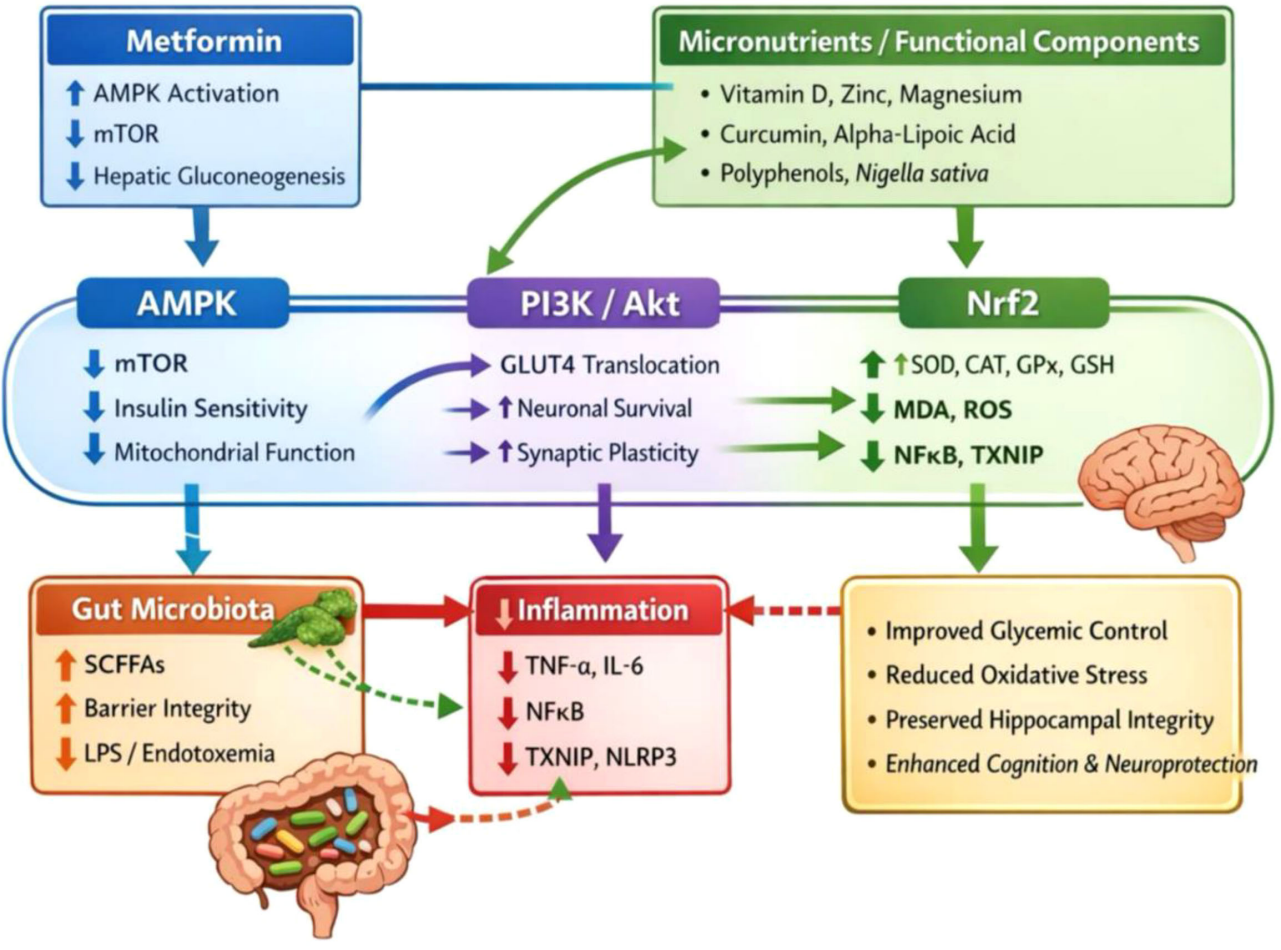
Integrated mechanistic pathways underlying micronutrient–metformin synergy in type 2 diabetes.

The collective evidence indicates that combined micronutrient and pharmacologic interventions produce complementary metabolic and neuroprotective effects in type 2 as summarized in **Table 13**. Most studies demonstrated activation of AMPK, PI3K/AKT, and BDNF signaling pathways, accompanied by reduced oxidative and inflammatory mediators. These molecular changes were strongly associated with improvements in glucose homeostasis, insulin sensitivity, and cognitive function as depicted by **Figure 6**. Natural compounds such as Ocimum gratissimum flavonoids, berberine, curcumin nanoparticles, vitamin D, vitamin E, zinc, and alpha-lipoic acid consistently enhanced antioxidant enzyme activity, restored GSH and SOD balance, and lowered MDA, IL-6, and TNF-α levels. Such biochemical modulation translated into better neuronal survival, enhanced synaptic plasticity, and preservation of hippocampal integrity. Moreover, a GRADE-assessed meta-analysis shows that functional components improve cardiovascular risk markers in patients with T2DM (61). This supports the view that micronutrient–metformin therapy provides indirect neurocognitive protection by reducing cardiovascular and inflammatory burden in addition to direct metabolic effects.

**Table 13:** Mechanistic insights of micronutrient and drug combinations in type 2 diabetes and neurocognitive dysfunction.

Mechanistic theme/intervention	Primary mechanism(s) of action	Biological effects/pathway modulation	Observed functional outcome	Reference
Environmental Enrichment (EE)	Gut–brain axis modulation, microbiota restoration	Decreased dysbiosis, decreased endotoxemia, decreased IL-6 and TNF-α, increased Muc2 expression	Improved insulin sensitivity, glucose metabolism, and cognition	(13)
Flavonoid-rich *Ocimum gratissimum* + Metformin	PI3K/AKT–GLUT4 activation, antioxidant signaling	Decreased MDA, TNF-α; increased SOD, BDNF, and hippocampal neuronal density	Neuroprotective synergy with improved learning and memory	(14)
Sildenafil + Metformin	PI3K/AKT–NO–cGMP regulation	Decreased IL-6, TNF-α, and NOS activity; increased glutathione preservation	Reduced neuropathic pain and oxidative stress	(6)
Zinc–FGF21 Axis (Smoker study)	Trace element–hormonal cross-talk	Increased FGF21, decreased ghrelin, modulated appetite regulation	Revealed link between trace elements and metabolic dysregulation	(15)
Carnosine Supplementation	Postprandial glucose modulation	Decreased glucose AUC, improved hepatic glucose regulation	Enhanced glucose handling and tolerance	(16)
Montelukast (Leukotriene antagonist)	Antioxidant and anti-inflammatory effects	Decreased MDA, BUN, and microalbuminuria; increased GSH	Improved renal antioxidant defense and function	(17a)
Memantine (NMDA antagonist)	TXNIP–NLRP3–IL-1β inhibition	Decreased ROS, IL-1β, and NLRP3; restored retinal integrity	Reduced oxidative injury and neuroinflammation	(60)
Vitamin D ± NAC	AMPK–ER stress suppression	Decreased ROS, CHOP, GRP78; increased insulin secretion and β-cell viability	Enhanced β-cell protection and glucose regulation	(18a)
Liposomal Berberine + Vildagliptin	AMPK/mTOR–autophagy activation	Increased Beclin-1 and LC3-II; decreased CHOP and JNK activation	Improved hepatic insulin signaling and reduced ER stress	(19)
EE + Metformin Co-treatment	BDNF/TrkB–PI3K–Akt–MAPK cascade	Increased BDNF and synaptic protein expression; decreased GSK3β activity	Enhanced neuronal survival and memory performance	(5)
Stress-induced Hyperglycemia (GAR study)	Dysregulated acute–chronic glucose balance	Increased oxidative load, increased mortality in high-FPG quartiles	Poor prognosis and recovery post-stroke (non-diabetics)	(20)
Resveratrol (SIRT2 activator)	SIRT2–glycolytic enzyme activation	Increased HK2, PKM2, and LDHA expression; decreased IGF1	Improved insulin sensitivity and ovarian metabolic function	(21)
AgNPs (Flacourtia cretica)	Antioxidant and enzyme inhibition	Decreased α-amylase and α-glucosidase activity; increased β-cell regeneration	Antidiabetic and pancreatic protective outcomes	(40)
Ascorbic Acid (Vitamin C)	HPA axis regulation, antioxidant modulation	Decreased corticosterone and lipid peroxidation; increased IL-10	Reduced stress-related inflammation and improved metabolism	(41)
Magnesium Supplementation	Cardiac autonomic regulation	Increased HRV and diastolic compliance; unchanged glycemia	Enhanced cardiac function independent of glucose levels	(42)
Rutaecarpine (Evodia rutaecarpa)	AMPK/ACC2–PI3K/AKT pathway	Decreased TC, TG, and LDL; increased HDL; decreased NF-κB	Improved lipid metabolism and insulin responsiveness	(21)
Tocotrienol-rich Fraction (TRF)	PDGF-C and antioxidant restoration	Increased SOD, GSH, and PDGF-C; decreased MDA	Enhanced neurovascular integrity and cognitive function	(43)
Zinc Sulfate Supplementation	Antioxidant and hematologic modulation	Increased ferritin, Hb, and MCV; decreased vitamin B12 and homocysteine	Improved iron metabolism and reduced cardiovascular risk	(44)
Curcumin/Zn Nanoparticles	MAPK/PI3K–Akt activation	Decreased amyloid-beta and tau phosphorylation; increased SOD, GPx	Improved learning and neuronal oxidative stability	(45)
Vitamin D3 + Rivastigmine	AMPK–PI3K–Akt–mTOR–BDNF signaling	Decreased Aβ42 and MDA; increased GSH and insulin	Restored insulin signaling and cognitive performance	(29)
Alpha-lipoic Acid + Metformin	Nrf2–HO-1/AMPK synergy	Decreased TNF-α, IL-6, and MDA; increased BDNF, PSD-95	Enhanced mitochondrial redox balance and memory outcomes	(39)
Nigella sativa Oil ± Metformin/Glimepiride	AMPK–IRS–Akt–GSK3β cascades regulation	Decreased Aβ42, phosphorylated Tau, IL-6, and TNF-α	Restored brain insulin signaling and improved cognition	(40)
Metformin Long-Term Use (Antagonistic Effect)	Nutrient depletion mechanism	Decreased vitamin B12; increased homocysteine, IL-6, TNF-α, and hsCRP	Worsened neuroinflammation and cognitive decline (antagonistic effect)	(41)

Several nutrients–drug combinations, including metformin with vitamin D_3_, alpha-lipoic acid, and curcumin nanoparticles, showed synergistic regulation of insulin signaling and oxidative defense, resulting in greater neurocognitive improvement compared with single treatments. A small subset of studies reported antagonistic interactions, notably with prolonged metformin therapy, where vitamin B12 depletion and homocysteine accumulation contributed to inflammation and cognitive decline.

The schematic illustrates how metformin primarily activates AMPK-dependent metabolic signaling to improve insulin sensitivity and suppress hepatic gluconeogenesis, while micronutrients and functional components modulate complementary pathways, including PI3K/Akt–mediated insulin and neuroplastic signaling, Nrf2-dependent antioxidant defense, and gut microbiota–inflammation interactions. Convergence of these pathways reduces oxidative stress and inflammatory burden, enhances mitochondrial and synaptic function, preserves hippocampal integrity, and collectively contributes to improved glycemic control and neurocognitive protection in type 2 diabetes.

The table summarizes key mechanistic insights into nutrient–drug interactions in type 2 diabetes. Most interventions demonstrated increased AMPK–PI3K–BDNF activation and decreased oxidative and inflammatory mediators, contributing to improved cognitive and metabolic outcomes.

However, long-term metformin use showed a clear antagonistic effect, marked by decreased vitamin B12 and increased homocysteine and inflammatory cytokines, emphasizing the need for concurrent micronutrient supplementation in chronic therapy. However, clinical evidence is constrained by small sample sizes, short follow-up durations, and heterogeneous study designs, limiting conclusions on long-term efficacy and safety and necessitating cautious, individualized application of micronutrient–metformin combinations. These findings bear important implications for global health policy, especially under frameworks such as the WHO Global Diabetes Compact (2023–2030) and the UN Decade of Action on Nutrition (2016–2025) (58, 62). Both initiatives emphasize integrative management of metabolic diseases through nutrition-sensitive interventions.

## Conclusion

This review demonstrates that integrating micronutrient supplementation within type 2 diabetes management provides a promising strategy for improving both metabolic and cognitive health. The collective evidence supports the role of nutrient drug synergy as an emerging frontier in diabetes care, offering a safe, accessible, and cost-effective adjunct to conventional therapies. The findings emphasize the importance of incorporating nutrition-based interventions into clinical guidelines and public health strategies, consistent with global initiatives such as the WHO Global Diabetes Compact and the UN Decade of Action on Nutrition. Strengthening micronutrient surveillance, personalized supplementation, and interdisciplinary diabetes management could substantially reduce disease burden and enhance long-term quality of life for affected populations. Future research should focus on dose optimization, mechanistic validation, and long-term clinical evaluation to support the development of standardized protocols for combined nutraceutical–pharmacologic therapy. Study designs should explicitly account for polypharmacy in individuals with type 2 diabetes by evaluating micronutrient–metformin combinations alongside commonly prescribed concurrent medications, including statins, antihypertensive agents, and lipid-lowering therapies, to enable safe and individualized interventions. In addition, long-term safety monitoring, patient adherence, and systematic adverse event reporting should be prioritized to enhance clinical translatability and inform policy integration of micronutrient monitoring and nutrition-based strategies within routine diabetes care frameworks.

## Limitations

Study Heterogeneity: The included studies varied widely in design, model type (preclinical and clinical), sample size, intervention duration, and outcome assessment, limiting cross-comparability thus, limiting Meta-analysis.

Non-standardized Measures: Cognitive and biochemical endpoints were assessed using diverse tools and biomarkers, reducing the ability to perform uniform comparisons or quantitative synthesis.

Incomplete Data Reporting: Several studies lacked detailed reporting of doses, participant characteristics, or statistical outcomes, which may have affected the accuracy of interpretation.

Publication Bias: A tendency toward reporting positive results may have overrepresented beneficial effects of micronutrient and metformin interventions.

Limited Randomized Clinical Evidence: Few large-scale or long-term randomized controlled trials were available, restricting the generalizability of findings to real-world clinical settings.

Unassessed Safety and Adherence: Most studies did not evaluate long-term safety, tolerability, or adherence factors, which are crucial for translating these interventions into routine practice.

Recommendations

1. Integrative Clinical Practice: Micronutrient supplementation particularly vitamin D, zinc, and antioxidant compounds should be integrated into standard diabetes management protocols to enhance glycemic control and neurocognitive health.
2. Clinical Research Priorities: Future trials should adopt longitudinal and multi-center designs to determine optimal dosing, safety profiles, and synergistic effects of combined micronutrient–metformin therapy.
3. Policy and Health Systems: Health ministries and diabetes programs should incorporate routine micronutrient screening and nutrition-based interventions into national diabetes care frameworks in line with the WHO Global Diabetes Compact.
4. Capacity Building: Strengthening laboratory infrastructure and clinical nutrition training is essential for translating emerging evidence into sustainable healthcare practice.
5. Public Health Implementation: Community-based awareness programs should emphasize dietary diversification and safe supplementation to prevent micronutrient deficiencies contributing to diabetes progression

## Glossary

AD: Alzheimer’s Disease
ACh: Acetylcholine
AChE: Acetylcholinesterase
Akt: Protein Kinase B
AMPK: AMP-Activated Protein Kinase
APP: Amyloid Precursor Protein
BACE-1: Beta-Site APP Cleaving Enzyme 1
BDNF: Brain-Derived Neurotrophic Factor
CAT: Catalase
CRP: C-Reactive Protein
CREB: cAMP Response Element-Binding Protein
EE: Environmental Enrichment
FPG: Fasting Plasma Glucose
G6Pase: Glucose-6-Phosphatase
GLUT4: Glucose Transporter Type 4
GPx: Glutathione Peroxidase
GSH: Reduced Glutathione
GSK3β: Glycogen Synthase Kinase 3 Beta
HbA1c: Glycated Hemoglobin
HDL-C: High-Density Lipoprotein Cholesterol
HFD: High-Fat Diet
HOMA-IR: Homeostatic Model Assessment of Insulin Resistance
HRV: Heart Rate Variability
IL-1β: Interleukin-1 Beta
IL-6: Interleukin-6
IL-10: Interleukin-10
iNOS: Inducible Nitric Oxide Synthase
IRS: Insulin Receptor Substrate
LDH: Lactate Dehydrogenase
LDL-C: Low-Density Lipoprotein Cholesterol
MAPK: Mitogen-Activated Protein Kinase
MDA: Malondialdehyde
MMSE: Mini-Mental State Examination
MoCA: Montreal Cognitive Assessment
mTOR: Mechanistic Target of Rapamycin
NAC: N-Acetylcysteine
NF-κB: Nuclear Factor Kappa B
NLRP3: NOD-, LRR-, and Pyrin Domain-Containing Protein 3
NQO1: NAD(P)H Quinone Dehydrogenase 1
Nrf2: Nuclear Factor Erythroid 2-Related Factor 2
OGTT: Oral Glucose Tolerance Test
PI3K: Phosphoinositide 3-Kinase
PPAR: Peroxisome Proliferator-Activated Receptor
PSD-95: Postsynaptic Density Protein 95
ROS: Reactive Oxygen Species
SIRT1: Sirtuin 1
SOD: Superoxide Dismutase
STZ: Streptozotocin
TBARS: Thiobarbituric Acid Reactive Substances
T2DM: Type 2 Diabetes Mellitus
TNF-α: Tumor Necrosis Factor Alpha
TXNIP: Thioredoxin-Interacting Protein
ZnSO₄: Zinc Sulfate
